# Effectiveness of eHealth Smoking Cessation Interventions: Systematic Review and Meta-Analysis

**DOI:** 10.2196/45111

**Published:** 2023-07-28

**Authors:** Yichen E Fang, Zhixian Zhang, Ray Wang, Bolu Yang, Chen Chen, Claudia Nisa, Xin Tong, Lijing L Yan

**Affiliations:** 1 Global Health Research Center Duke Kunshan University Kunshan China; 2 Department of Global Health, School of Public Health Wuhan University Wuhan China; 3 Division of Social Sciences Duke Kunshan University Kunshan China; 4 Data Science Research Center Duke Kunshan University Kunshan China; 5 Duke Global Health Institute Duke University Durham, NC United States; 6 Institute for Global Health and Development Peking University Beijing China

**Keywords:** smoking cessation, systematic review, meta-analysis, electronic health, mobile health, eHealth, smoking, development, technology-assisted, effectiveness, screening, data extraction, user, design, mobile phone

## Abstract

**Background:**

Rapid advancements in eHealth and mobile health (mHealth) technologies have driven researchers to design and evaluate numerous technology-based interventions to promote smoking cessation. The evolving nature of cessation interventions emphasizes a strong need for knowledge synthesis.

**Objective:**

This systematic review and meta-analysis aimed to summarize recent evidence from randomized controlled trials regarding the effectiveness of eHealth-based smoking cessation interventions in promoting abstinence and assess nonabstinence outcome indicators, such as cigarette consumption and user satisfaction, via narrative synthesis.

**Methods:**

We searched for studies published in English between 2017 and June 30, 2022, in 4 databases: PubMed (including MEDLINE), PsycINFO, Embase, and Cochrane Library. Two independent reviewers performed study screening, data extraction, and quality assessment based on the GRADE (Grading of Recommendations, Assessment, Development, and Evaluations) framework. We pooled comparable studies based on the population, follow-up time, intervention, and control characteristics. Two researchers performed an independent meta-analysis on smoking abstinence using the Sidik-Jonkman random-effects model and log risk ratio (RR) as the effect measurement. For studies not included in the meta-analysis, the outcomes were narratively synthesized.

**Results:**

A total of 464 studies were identified through an initial database search after removing duplicates. Following screening and full-text assessments, we deemed 39 studies (n=37,341 participants) eligible for this review. Of these, 28 studies were shortlisted for meta-analysis. According to the meta-analysis, SMS or app text messaging can significantly increase both short-term (3 months) abstinence (log RR=0.50, 95% CI 0.25-0.75; *I^2^*=0.72%) and long-term (6 months) abstinence (log RR=0.77, 95% CI 0.49-1.04; *I^2^*=8.65%), relative to minimal cessation support. The frequency of texting did not significantly influence treatment outcomes. mHealth apps may significantly increase abstinence in the short term (log RR=0.76, 95% CI 0.09-1.42; *I^2^*=88.02%) but not in the long term (log RR=0.15, 95% CI −0.18 to 0.48; *I^2^*=80.06%), in contrast to less intensive cessation support. In addition, personalized or interactive interventions showed a moderate increase in cessation for both the short term (log RR=0.62, 95% CI 0.30-0.94; *I^2^*=66.50%) and long term (log RR=0.28, 95% CI 0.04-0.53; *I^2^*=73.42%). In contrast, studies without any personalized or interactive features had no significant impact. Finally, the treatment effect was similar between trials that used biochemically verified or self-reported abstinence. Among studies reporting outcomes besides abstinence (n=20), a total of 11 studies reported significantly improved nonabstinence outcomes in cigarette consumption (3/14, 21%) or user satisfaction (8/19, 42%).

**Conclusions:**

Our review of 39 randomized controlled trials found that recent eHealth interventions might promote smoking cessation, with mHealth being the dominant approach. Despite their success, the effectiveness of such interventions may diminish with time. The design of more personalized interventions could potentially benefit future studies.

**Trial Registration:**

PROSPERO CRD42022347104; https://www.crd.york.ac.uk/prospero/display_record.php?RecordID=347104

## Introduction

### Background

Smoking is a major risk factor for cancer and cardiovascular, respiratory, and many other chronic diseases worldwide [1]. In addition to the significant health burden it imposes, smoking also incurs massive economic costs. The United States alone lost a staggering US $864.5 billion in 2020 due to this issue [2], whereas limited-income countries, such as China, with higher smoking prevalence, face similar challenges [3]. Smoking cessation is crucial for minimizing mortality risk and improving quality of life [4]. Hence, finding effective ways to promote smoking cessation among smokers continues to be a vital public health goal. However, traditional smoking cessation services such as counseling can be expensive [5,6] and poorly received because of factors such as patients’ lack of time or reluctance to seek cessation services in clinical settings [7]. These challenges necessitate the development of cost-effective models for reducing tobacco consumption.

eHealth technologies, such as websites, mobile apps, and SMS text messages, have emerged as low-cost accessible interventions. Many of these technologies offer interactive experiences to users [8], which can enhance patient adherence to cessation services [9]. As such, they are ideal tools for revolutionizing health care [10] and promoting smoking cessation for diverse user groups, including ordinary daily smokers and pregnant women who wish to quit for their children’s well-being.

Numerous studies have investigated the effectiveness of eHealth cessation interventions over the past decade, but their findings have been inconsistent [11]. For example, a systematic review of 108 studies in 2018 found evidence suggesting that web-based and mobile health (mHealth) interventions could moderately increase abstinence rates, whereas computer-assisted interventions did not show the same effect [12]. Another 2019 systematic review of 26 studies indicated that automated text messaging interventions were more effective than minimal smoking cessation support, whereas the effectiveness of mobile apps on abstinence remains unclear [13]. However, these systematic reviews have some notable limitations. Recent reviews only evaluated abstinence as the outcome variable and did not include other outcomes such as cigarette consumption [14]. In addition, the reviews did not differentiate between self-reported versus biochemically verified abstinence. Another drawback is that previous reviews primarily synthesized evidence from high-income countries, which may not be generalizable to low- and middle-income countries [15]. Finally, the most recent review, which covered the entire eHealth intervention landscape, only included studies published until 2017 [12]. Since then, the use of eHealth has skyrocketed and many new studies have been published.

### Objectives

With the rapid development of eHealth technologies [16], smoking cessation interventions are constantly evolving and are dynamic in nature, encompassing both delivery channels and intervention materials. Therefore, this systematic review aimed to (1) summarize recent evidence (from 2017 to mid-2022) on the effectiveness of eHealth-based smoking cessation interventions, grouped by treatment characteristics, study population, and outcome verification, and (2) assess important nonabstinence outcome indicators such as cigarette consumption and user satisfaction via narrative synthesis.

## Methods

This study was designed and reported in accordance with the PRISMA (Preferred Reporting Items for Systematic Reviews and Meta-Analyses) statement [17]. A detailed protocol containing the objectives and methods of this systematic review is registered in PROSPERO (2022; CRD42022347104).

### Search Strategy

A systematic search was performed across 4 electronic databases—PubMed (including MEDLINE), PsycINFO, Embase, and Cochrane Library—to gather studies published between 2017 and mid-2022. The search strategy was first created for PubMed using a combination of keywords and Medical Subject Heading terms. To make the search more precise, keywords were mapped to Medical Subject Heading terms where possible. We later applied the search strategy to other databases, namely PsycINFO, Embase, and the Cochrane Library, using their own thesaurus terms and advanced search features. The search terms were classified into four categories: (1) smoking cessation—the theme of the intervention; (2) device—the device used to carry out the intervention; (3) intervention channel—the specific approach used to engage the participants; and (4) randomized controlled trials (RCTs)—the study design. Each database was searched accordingly. The search strategy for PubMed is shown in Table 1. Multimedia Appendix 1 [18] documents search terms for all databases. The search results were limited to studies published in English from January 1, 2017, to June 30, 2022, given that previous review papers on eHealth smoking cessation interventions primarily collected studies published before 2017 [12].

**Table 1:** Search strategy: PubMed key terms.

Topics	Key terms
Smoking cessation	“Smoking Cessation”(MeSH^a^ terms)
Device	(“Cell Phone”[MeSH terms] OR “smartphone”[MeSH terms] OR “computers”[MeSH terms] OR “Computers, Handheld”[MeSH terms])
Intervention channel	(“Online Systems”[MeSH terms] OR “Technology”[MeSH terms] OR “Social Media”[MeSH terms] OR “Mobile Applications”[MeSH terms] OR “Text Messaging”[MeSH terms] OR “telemedicine”[MeSH terms] OR “Internet-Based Intervention”[MeSH terms] OR “multimedia”[MeSH terms] OR “Electronic Mail”[MeSH terms])
RCT^b,c^	([Randomized controlled trial(Pt)] OR [controlled clinical trial(Pt)] OR [randomized(tiab) OR randomized(tiab)] OR [placebo(tiab)] OR [drug therapy(sh)] OR [randomly(tiab)] OR [trial(tiab)] OR [groups(tiab)]) NOT (animals[mh] NOT humans[mh])

^a^MeSH: Medical Subject Heading.

^b^RCT: randomized controlled trial.

^c^Key terms for RCTs were retrieved from McGill Library [18].

### Eligibility Criteria

#### Population

The study population included adults (aged ≥18 years) who were current smokers during enrollment in the study. We were interested in investigating the effectiveness of cessation interventions only on cigarette smoking.

#### Intervention

Studies reporting eHealth-based smoking cessation interventions, defined as interventions delivered through mobile-based, web-based, computer-based, portable device-based, and social media–based channels, were included. The intervention content may consist of educational readings, videos, and counseling based on various therapies; text messaging; social media; and even biochemical testing (eg, carbon monoxide checkers). Interventions were then classified into the following 3 groups under the broader category of eHealth interventions: web-based, mHealth (SMS text messages and apps), or computer-assisted interventions. Web-based interventions refer to cessation services available on websites, whereas mHealth interventions are defined as any cessation materials delivered through mobile phones. Finally, computer-assisted interventions refer to cessation services that are accessible via computers. eHealth intervention can either be delivered in a stand-alone setting or as an adjunct to other therapies. Interventions were considered *personalized or interactive* if the intervention content was tailored to each participant, based on his or her response or ability to offer interactive experience through live feedback.

#### Control or Comparator

Studies that included placebo or control interventions, non-eHealth interventions, or no interventions as controls were included. Placebo or control interventions may consist of delivering less related content through electronic channels, such as a reduced version of an mHealth cessation app. Non-eHealth interventions may include smoking cessation content provided in nonelectronic media, such as self-help cessation materials. This systematic review included only studies with at least 1 control group.

#### Outcomes

Studies reporting biochemically verified or self-reported abstinence were measured at ≥3 months of follow-up. Other outcomes, such as reduction in cigarette consumption and adherence to the intervention, as measured by the satisfaction rate, were also recorded when available but were not mandatory.

#### Study Design

Only RCTs were included in this review, including both full-scale RCTs and pilot RCTs. Conference abstracts were excluded from the study.

### Exclusion Criteria

Studies were excluded if they (1) included people using smokeless tobacco products or e-cigarettes, (2) only used eHealth technology during the recruitment of participants and not as part of the intervention, and (3) had a follow-up period shorter than 3 months.

### Study Selection

Two independent reviewers (YEF and ZZ) screened titles and abstracts for potential inclusion. A relatively good interrater reliability was achieved (proportionate agreement=81%, Cohen κ=0.61). The same pair of reviewers also independently performed a full-text review after screening for final inclusion. Any conflicts between the 2 reviewers were discussed in the presence of a third author (selected from the author list, either RW or BY), who contributed to the final consensus. The study selection process was performed using the Covidence workflow platform.

### Data Extraction

Two reviewers (YEF and ZZ) independently performed data extraction using the same data extraction template with multiple categories for detailed information input on the Covidence platform [19]. Extracted data included the following: (1) study information (country of study, trial registration, funding sources, and declarations of interest); (2) study participants (inclusion or exclusion criteria, population characteristics, and sample size); (3) intervention and control details; (4) theoretical framework; (5) outcome measurements; and (6) key study results (abstinence rate, reduction in cigarette consumption, and satisfaction rate at ≥3 month follow-up). Any conflicts between the 2 authors were discussed between the reviewers or in the presence of a third author (either RW or BY) for final consensus.

### Risk of Bias (Quality) Assessment

Quality assessment was based on the GRADE (Grading of Recommendations, Assessment, Development, and Evaluations) framework for quality assessment [17]. Two reviewers (YEF and ZZ) first conducted the risk of bias assessment independently under the guidance of the Cochrane Handbook for Systematic Reviews of Interventions [20] and the Cochrane Tobacco Addiction Group. Two reviewers assessed the risk of bias for each included study via five prespecified domains using version 2 of the Cochrane risk-of-bias tool for randomized trials (RoB 2) [21]: (1) bias arising from the randomization process, (2) bias due to deviations from intended interventions, (3) bias due to missing outcome data, (4) bias in measurement of the outcome, and (5) bias in selection of the reported result. After data extraction, each reviewer judged each domain as having low, high, or some concern. Disagreements were resolved between the 2 authors in the presence of a third author. The certainty of the evidence was rated as very low, low, moderate, or high based on the risks of bias, imprecision, inconsistency, indirectness, and publication bias.

### Synthesis of Results

The primary outcome of this systematic review was to evaluate the impact of eHealth-based smoking cessation interventions on the abstinence rate measured at ≥3-month follow-up via self-report or biochemical verification. Short- and long-term abstinence was defined as the abstinence result measured at the 3- and 6-month follow-ups, respectively. The measurements were 7-day point prevalence abstinence (PPA), 30-day PPA, or prolonged PPA. These measurements were used interchangeably in this review because there is evidence suggesting that such data handling does not significantly affect the results [22]. Only intention-to-treat analysis data were selected. All initially randomized participants were included, and any missing data caused by withdrawal were considered smokers based on the Cochrane Tobacco Group guidelines [23]. Nonabstinence outcomes were not subjected to meta-analysis because of the limited number of studies reporting the statistics and variation in outcome measurement standards. Subsequently, the reductions in cigarette consumption and satisfaction rates were narratively synthesized.

For the primary outcome of abstinence rate, dichotomous data on quit or smoking participants’ numbers in either the treatment or control groups at follow-ups were entered into Stata 17 software [24] to calculate the log risk ratio (RR). The included studies were stratified into different subgroups based on their study participants, eHealth interventions or controls, and outcome verification for comparable results in the meta-analysis. Where 2 or more studies were deemed comparable, we performed a meta-analysis to calculate the combined effects of the interventions on the abstinence rate. For studies that were not included in the meta-analysis, we summarized the abstinence outcomes for each study. Considering the potential treatment effect heterogeneity, differences in trial size, and the limited number of included studies, this study used the Sidik-Jonkman random-effects model method to pool log RRs and 95% CIs calculated for the abstinence outcome [25]. Heterogeneity was assessed using *I*^2^ statistic, given its robustness with small sample sizes. Publication bias was assessed using funnel plots.

## Results

### Study Selection

**Figure 1:**
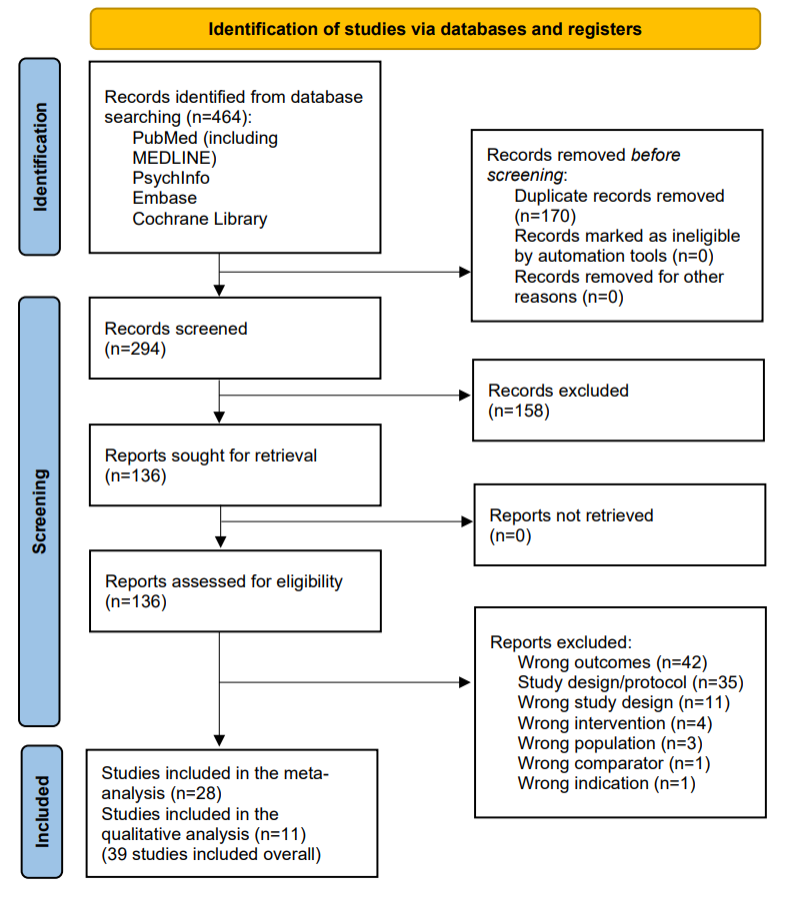
PRISMA (Preferred Reporting Items for Systematic Reviews and Meta-Analyses) diagram of searching and screening process.

After removing duplicates, we identified a total of 464 studies in the initial database search. After screening and full-text assessments, 39 studies were deemed eligible for this review, of which 28 were included in the meta-analysis. Figure 1 shows the PRISMA flowchart, which illustrates the process of study selection and rationales for exclusion during full-text assessments.

### Study Characteristics

#### Overview

All included studies were RCTs (31/39, 80%) or pilot RCTs (8/39, 21%) published between 2017 and 2022. The key characteristics of the included studies are summarized in Table 2. Most studies were conducted in high-income economies (28/39, 72%; ie, the United States, the United Kingdom, France, Switzerland, Spain, Argentina, Hong Kong, and Japan). However, a relatively substantial number of studies were found in low- and middle-income countries or regions (11/39, 28%) defined by the World Bank [26] (ie, China, Thailand, India, Brazil, Turkey, and Vietnam).

**Table 2:** Summary of characteristics of included studies (n=39).

Characteristics			Meta-analysis (n=28), n (%)		Non–meta-analysis (n=11), n (%)		Total studies (n=39), n (%)
**Intervention**							
	Web-based	1 (4)		0 (0)		1 (3)	
	mHealth^a^	25 (89)		5 (45)		30 (77)	
	Multiplatform	2 (7)		6 (55)		8 (21)	
**Country**							
	High-income countries or regions	19 (68)		9 (82)		28 (72)	
	Low- and middle-income countries or regions	9 (32)		2 (18)		11 (28)	
**Delivery**							
	Personalized or interactive	19 (68)		10 (91)		29 (74)	
	Not personalized or interactive	9 (32)		1 (9)		10 (26)	
**Participants**							
	Adult smokers only with intention to quit	18 (64)		8 (73)		26 (67)	
	Adult smokers with or without intention to quit	0 (0)		1 (9)		1 (3)	
	Pregnant smokers	5 (18)		0 (0)		5 (13)	
	Smokers with mental disorders	3 (11)		0 (0)		3 (77)	
	Other susceptible individuals	2 (7)		2 (18)		4 (10)	
**eHealth role**							
	eHealth as primary intervention	23 (82)		11 (100)		34 (87)	
	eHealth as adjunct intervention	5 (18)		0 (0)		5 (13)	
**Theoretical framework**							
	Cognitive behavioral therapy	1 (4)		2 (18)		3 (8)	
	Mindfulness (acceptance and commitment therapy)	4 (14)		2 (18)		6 (15)	
	Social cognitive theory	3 (11)		1 (9)		4 (10)	
	Multitheories	4 (14)		0 (0)		4 (10)	
	Other	6 (21)		2 (18)		8 (21)	
	Not stated	10 (36)		4 (36)		14 (36)	
**Abstinence verification**							
	Self-reported	10 (36)		7 (64)		17 (44)	
	Biochemically verified	18 (64)		4 (36)		22 (56)	
**Reported outcome other than abstinence (percentage may not add up to 100%)**							
	Cigarette consumption	10 (36)		4 (36)		14 (36)	
	User satisfaction	15 (54)		4 (36)		19 (38)	
**Longest reported length of follow-up**							
	3 months	10 (36)		2 (18)		12 (31)	
	6 months (including late pregnancy)	14 (50)		5 (45)		19 (49)	
	12 months	4 (14)		4 (36)		8 (21)	

^a^mHealth: mobile health.

#### Participants

A total of 37,341 participants from 39 studies were included in this review. The sample size per study varied from 49 to 8000 participants. Most participants (26/39, 67% of studies) were nonclinical adult smokers who intended to quit smoking. The term *intention-to-quit* refers to smokers who were willing to quit smoking upon recruitment. Other study participants included adult smokers who did not necessarily intend to quit smoking and were recruited in occupational settings (1/39, 3%), pregnant smokers (5/39, 13%), and smokers with mental disorders (3/39, 8%). It is worth noting that studies involving pregnant smokers (5/39, 13%) included participants aged ≥16 years. The average age of pregnant women ranged from 26.6 to 28 years, suggesting that most recruited participants were adults. Therefore, we included these studies in our analysis to provide a comprehensive overview of eHealth-based cessation interventions. Other susceptible populations identified included smokers from lower socioeconomic backgrounds, patients currently with tuberculosis, and hospitalized patients in clinical settings (4/39, 10%; Table 2).

#### Interventions

Notably, most studies reported the use of mHealth interventions (30/39, 77%) comprising SMS text messages or mobile apps. For clarity, we henceforth refer SMS text messages and apps that only provide messaging services as *SMS or app text messaging*. In addition to mHealth, 1 study used web-based intervention (1/39, 3%), and 8 studies adopted mixed approaches (8/39, 21%), where mHealth and web-based channels were both used in the intervention packages. Most eHealth interventions (34/39, 87%) were delivered as primary interventions. Over two-thirds (29/39, 74%) of the interventions involved some degree of personalization through tailored intervention materials based on user feedback or by providing interactive experiences. More than one-third (14/39, 36%) of the studies did not specify a theoretical framework. The theoretical frameworks mentioned were primarily acceptance and commitment therapy, social cognitive theory, cognitive and behavioral therapy, or mixed theories.

#### Outcomes

More than half (22/39, 56%) of the studies adopted biochemical verification through carbon monoxide testing or cotinine testing for PPA measurements. The remaining studies used self-reported abstinence data. The duration of follow-up ranged from 3 months (12/39, 31%) to 6 months or before delivery (19/39, 49%) to 12 months (8/39, 21%). Apart from the primary outcome of smoking abstinence, 13 (13/39, 33%) studies reported changes in cigarette consumption, whereas 19 (19/39, 38%) studies reported user satisfaction after intervention.

### Risk of Bias in Included Studies

**Figure 2:**
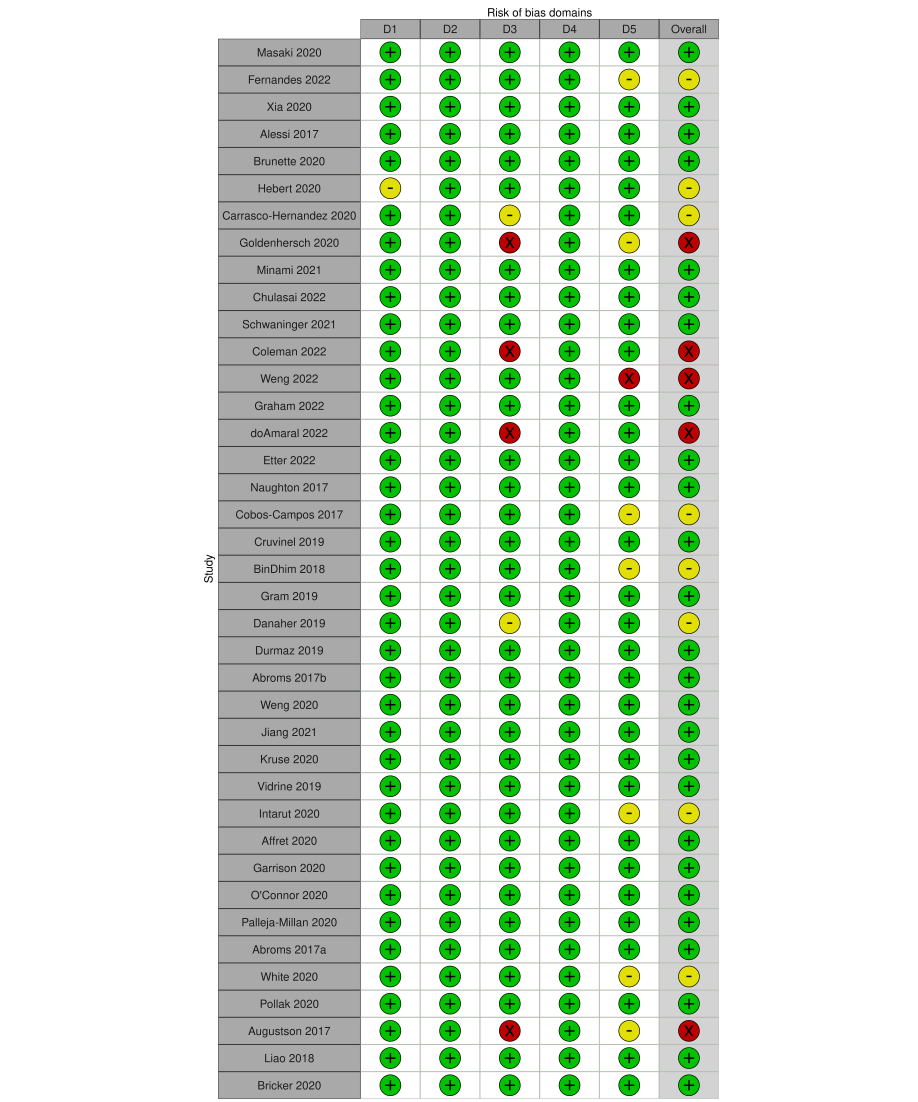
Risk of bias graph based on review authors’ judgments across all included studies (n=39) [27-65]. Risk of bias domains: D1: bias arising from the randomization process; D2: bias due to deviations from intended intervention; D3: bias due to missing outcome data; D4: bias in the measurement of the outcome; D5: bias in selection of the reported result. Judgement: ⊕: high, 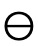: some concerns, ⊗: low.

All included studies underwent a risk of bias assessment based on the guidelines suggested by the Cochrane Handbook for Systematic Reviews of Interventions [20]. Bias in measurement of the outcome was rated as low risk because the Cochrane Tobacco Addiction Group stated that blinding participants made cessation interventions impossible [13]. In summary, we evaluated 26 (26/39, 67%) studies to be at low risk of bias (considered low risk of bias for all domains), 8 (8/39, 21%) studies with some concerns (considered with some concerns for at least 1 domain, but with no judgments of high risk), and 5 (5/39, 13%) studies at high risk (considered high risk of bias in at least 1 domain). The risk of bias per domain is shown in Figure 2. Incomplete outcome data was the primary cause of high risk of bias (4/39, 10%). It is worth noting that 33% (13/39) studies reported a high attrition rate (>20%), but 9 were deemed to have a low risk of bias or some concerns because there was no evidence for differential missing data.

### Meta-Analysis of Smoking Abstinence Results (Primary Outcome)

#### Overview

A total of 28 studies were included in the meta-analysis because of similarities in the target population, intervention, control, and outcomes. To ensure comparability among the studies in the meta-analysis, they were divided into short-term (3-month follow-up) and long-term (6-month follow-up) studies involving general adult smokers (19/28, 69%). The results are presented in tabular format to facilitate the presentation of more information, and all forest plots are available in Multimedia Appendix 2 [38-65]. Within each follow-up category, the studies were grouped based on the type of intervention and control (Table 3): (1) high-frequency SMS or app text messaging versus low-frequency SMS or app text messaging; (2) SMS or app text messaging versus minimal cessation support (including self-help materials and standard practice); (3) mHealth app versus less intensive smoking cessation support (including existing cessation services or a mobile app with fewer functions); and (4) mHealth app + psycho or pharmacological therapy versus psycho or pharmacological therapy alone. In addition, we conducted exploratory analyses by pooling the same groups of studies based on personalization or interactive level (personalized or interactive vs nonpersonalized or interactive) or outcome verification types (biochemically verified vs self-reported; Table 4). For studies targeting special populations (10/28, 36%), we pooled the abstinence results of studies targeting the same population only by participant characteristics. Finally, detailed information on each study included in the meta-analysis is provided in Multimedia Appendix 3 [38-65].

**Table 3:** Summary of eHealth intervention effects on abstinence by intervention type and follow-up, based on GRADE (Grading of Recommendations, Assessment, Development, and Evaluations) guidelines.

Outcome and follow-up				Summary of the effect		Number of participants and studies		Quality of the evidence (GRADE)^a^		Summary for intervention
**Smokers with intention to quit (by follow-up)**										
	**High-frequency SMS or app text messaging versus low-frequency SMS or app text messaging**									
		3 months	Log RR^b^=−0.01, 95% CI −0.25 to 0.28; *I*^2^=38.77%; *Little or no increase*^c^		8958 participants; 2 studies		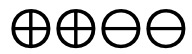 ^d,e^; Low		May make little or no increase on cessation	
		6 months	Log RR=0.00, 95% CI −0.07 to 0.08; *I*^2^=0.46%; *Little or no increase*		8958 participants; 2 studies		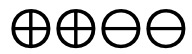 ^d,e^; Low		May make little or no increase on cessation	
	**SMS or app text messaging versus minimal smoking cessation support**									
		3 months	Log RR=0.50, 95% CI 0.25 to 0.75; *I*^2^=0.72%; *Moderate increase*		1367 participants; 5 studies		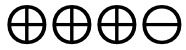 ^d^; Moderate		Probably increase cessation moderately	
		6 months	Log RR=0.77, 95% CI 0.49 to 1.04; *I*^2^=8.65%; *Important increase*		1153 participants; 3 studies		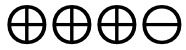 ^d^; Moderate		Probably increase cessation significantly	
	**mHealth^g^ app versus less intensive smoking cessation support**									
		3 months	Log RR=0.76, 95% CI 0.09 to 1.42; *I*^2^=88.02%; *Important increase*		1167 participants; 4 studies		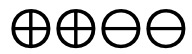 ^d,f^; Low		May increase cessation significantly	
		6 months	Log RR=0.15, 95% CI −0.18 to 0.48; *I*^2^=80.06%; *Little or no increase*		9360 participants; 6 studies		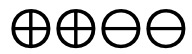 ^e,f^; Low		May make little or no increase on cessation	
	**mHealth app + psycho or pharmacological therapy versus psycho or pharmacological therapy**									
		6 months	Log RR=0.25, 95% CI −0.18 to 0.67; *I*^2^=16.91%; *Little or no increase*		340 participants; 2 studies		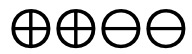 ^e,f^; Low		May make little or no increase on cessation	
**Smokers of special population (any follow-up)**										
	Adult smokers with mental disorders			Log RR=−0.25, 95% CI −1.92 to 1.42; *I*^2^=72.32%; *Little or no increase*		813 participants; 3 studies		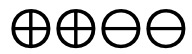 ^e,f^; Low		May make little or no increase on cessation
	Hospitalized adult smokers			Log RR=1.00, 95% CI 0.22 to 1.78; *I*^2^=3.45%; *Important increase*		466 participants; 2 studies		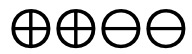 ^d,e^; Low		May increase cessation significantly
	Pregnant smokers (including adolescents)			Log RR=0.34, 95% CI −0.01 to 0.68; *I*^2^=25.84%; *Little or no increase*		2319 participants; 5 studies		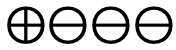 ^e,h^; Very low		May make little or no increase on cessation

^a^GRADE Working Group grades of evidence. 
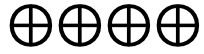
 High quality: The authors have a lot of confidence that the true effect is similar to the estimated effect. 
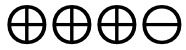
 Moderate quality: The authors believe that the true effect is probably close to the estimated effect. 
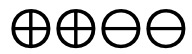
 Low certainty: The true effect might be markedly different from the estimated effect. Very low certainty: The true effect is probably markedly different from the estimated effect [66].

^b^RR: risk ratio.

^c^The italicization serves as an abstract description of the effect size based on the 95% CI: 95% CI crosses 0=little or no increase, 95% CI does not cross 0 nor 1=moderate increase, and 95% CI does not cross 0 but cross 1=important increase.

^d^Downgraded 1 level for significant risk of bias: one study was rated as high risk of bias (2 unclear risk of bias count as one high risk of bias).

^e^Downgraded 1 level for imprecision: CIs encompass both clinically significant harm and clinically significant benefit, or fewer than 500 participants overall.

^f^Downgraded 1 level of inconsistency: considerable unexplained statistical heterogeneity (*I*^2^>50%).

^g^mHealth: mobile health.

^h^Downgraded 2 levels for serious risk of bias: 2 or more studies rated as high risk of bias (2 unclear risk of bias count as one high risk of bias).

**Table 4:** Summary of exploratory analyses on eHealth intervention effects on abstinence by personalization or interactive level or outcome verification, based on GRADE (Grading of Recommendations, Assessment, Development, and Evaluations) guidelines.

Outcome verification and follow-up				Summary of the effect		Number of participants and studies		Quality of the evidence (GRADE)^a^		Summary for intervention
**Smokers with intention to quit (by follow-up)—personalization or interactive level**										
	**3 months (short-term)**									
		Personalized or interactive	Log RR^b^=0.62, 95% CI 0.30 to 0.94; *I*^2^=66.50%; *Moderate increase*^c^		2701 participants; 8 studies		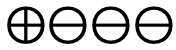 ^d,e^; Very low		May increase cessation moderately (true effect is probably markedly different)	
		Not personalized or interactive	Log RR=0.17, 95% CI −0.21 to 0.54; *I*^2^=67.39%; *Little or no increase*		8791 participants; 3 studies		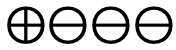 ^e,f,g^; Very low		May make little or no increase on cessation (true effect is probably markedly different)	
	**6 months (long-term)**									
		Personalized or interactive	Log RR=0.28, 95% CI 0.04 to 0.53; *I*^2^=73.42%**;** *Moderate increase*		10,695 participants; 9 studies		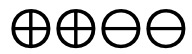 ^e,f^; Low		May increase cessation moderately	
		Not personalized or interactive	Log RR=0.23, 95% CI −0.26 to 0.72; *I*^2^=82.52%*; Little or no increase*		9116 participants; 4 studies		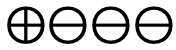 ^e,f,g^; Very low		May make little or no increase on cessation (true effect is probably markedly different)	
**Smokers with intention to quit (by follow-up)—verification**										
	**3 months (short-term)**									
		Biochemically verified results	Log RR=0.45, 95% CI 0.15 to 0.74; *I*^2^=21.39%; *Moderate increase*		1375 participants; 4 studies		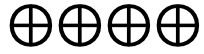 ; High		Increase cessation moderately	
		Self-reported results	Log RR=0.56, 95% CI 0.15 to 0.96; *I*^2^=87.88%; *Moderate increase*		10,117 participants; 7 studies		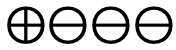 ^d,e,g^; Very low		May cessation moderately (true effect is probably markedly different)	
	**6 months (long-term)**									
		Biochemically verified results	Log RR=0.26, 95% CI −0.02 to 0.54; *I*^2^=34.82%; *Little or no increase*		2195 participants; 7 studies		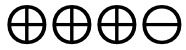 ^f,g^; Low		May make little or no increase on cessation	
		Self-reported results	Log RR=0.31, 95% CI −0.05 to 0.68; *I*^2^=95.06%; *Little or no increase*		17,616 participants; 6 studies		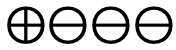 ^e,f,g^; Very low		May make little or no increase on cessation (true effect is probably markedly different)	

^a^GRADE Working Group grades of evidence. 
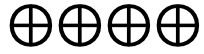
 High quality: The authors have a lot of confidence that the true effect is similar to the estimated effect. 
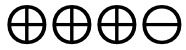
 Moderate quality: The authors believe that the true effect is probably close to the estimated effect. 
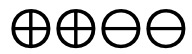
 Low certainty: The true effect might be markedly different from the estimated effect. Very low certainty: The true effect is probably markedly different from the estimated effect [66].

^b^RR: risk ratio.

^c^The italicization serves as an abstract description of the effect size based on the 95% CI: 95% CI crosses 0=little or no increase, 95% CI does not cross 0 nor 1=moderate increase, and 95% CI does not cross 0 but cross 1=important increase.

^d^Downgraded 2 levels for serious risk of bias: 2 or more studies rated as high risk of bias (2 unclear risk of bias count as one high risk of bias).

^e^Downgraded 1 level of inconsistency: considerable unexplained statistical heterogeneity (*I*^2^>50%).

^f^Downgraded 1 level for significant risk of bias: one study was rated as high risk of bias (2 unclear risk of bias count as one high risk of bias).

^g^Downgraded 1 level for imprecision: CIs encompass both clinically significant harm and clinically significant benefit, or fewer than 500 participants overall.

#### High-Frequency SMS or App Text Messaging and Low-Frequency SMS or App

Only 2 studies have compared high-frequency SMS or app text messaging with low-frequency SMS or app text messaging. When pooled, no statistically significant difference between the intervention and control outcomes was found in the short-term (log RR=−0.01, 95% CI −0.25 to 0.28; *I^2^*=38.77%) or long-term (log RR=0.00, 95% CI −0.07 to 0.08; *I^2^*=0.46%).

#### SMS or App Text Messaging Versus Minimal Smoking Cessation Support

In total, 5 studies compared the abstinence results of SMS or app text messaging with minimal smoking cessation support in the short term, and 3 reported long-term results (with an overlap between the studies). After pooling, a significant increase was found with moderate certainty in short-term abstinence in the intervention group (log RR=0.50, 95% CI 0.25-0.75; *I^2^*=0.72%). The effect was even more significant in the long-term follow-up (log RR=0.77, 95% CI 0.49-1.04; *I^2^*=8.65%).

#### mHealth App Versus Less Intensive Smoking Cessation Support

Among the studies included in the meta-analysis, 8 compared the mHealth app with less intensive smoking cessation support, with 4 reporting short-term results and 6 reporting long-term results (with overlap between the studies). The pooled abstinence outcome suggests that mHealth apps may have a significant short-term effect on abstinence for intervention (log RR=0.76, 95% CI 0.09 to 1.42; *I^2^*=88.02%), whereas no significant effect was found in the long term (log RR=0.15, 95% CI −0.18 to 0.48; *I^2^*=80.06%).

#### mHealth App + Psycho or Pharmacological Therapy Versus Psycho or Pharmacological Therapy Alone

Only long-term effects on abstinence were collected for studies that compared the mHealth app plus psycho or pharmacological therapy with psycho or pharmacological therapy alone (2/28, 7%). The difference in the abstinence outcome was not significant (log RR=0.25, 95% CI −0.18 to 0.67; *I^2^*=16.91%).

#### Personalized or Interactive Versus Not Personalized or Interactive

The same studies pooled in the subgroup analysis by intervention type in the short- and long-term were also pooled in the exploratory analyses by personalization or interaction level (Table 4). Compared with the studies with no personalization or interactive features that yielded nonsignificant results (short-term log RR=0.17, 95% CI −0.21 to 0.54; *I^2^*=67.39%; long-term log RR=0.23, 95% CI −0.26 to 0.72; *I^2^*= 82.52%), those that offered a certain level of personalization or interactive content reached moderate increases in abstinence both in the short term (log RR=0.62, 95% CI 0.30-0.94; *I^2^*= 66.50%) and long-term (log RR=0.28, 95% CI 0.04-0.53; *I^2^*=73.42%).

#### Biochemically Verified Outcomes Versus Self-Reported Outcomes

In the second exploratory analysis, we compared the intervention effect between studies that adopted biochemically verified and self-reported results. Studies that used biochemical verification for abstinence found a moderate increase in cessation in the short term (log RR=0.45, 95% CI 0.15-0.74; *I^2^*=21.39%), which is similar to the studies that used self-reported results (log RR=0.56, 95% CI 0.15 to 0.96; *I*^2^=87.88%). For long-term effects, neither of the 2 groups of studies achieved significant results (biochemical verification, log RR=0.26, 95% CI −.02 to 0.54; *I^2^*=34.82%; self-reported, log RR=0.31, 95% CI −0.05 to 0.68; *I^2^*=95.06%). Meanwhile, the estimates in the 2 exploratory analyses should be interpreted with caution given the substantial statistical heterogeneity.

#### eHealth Interventions Targeting Specific Populations

**Figure 3:**
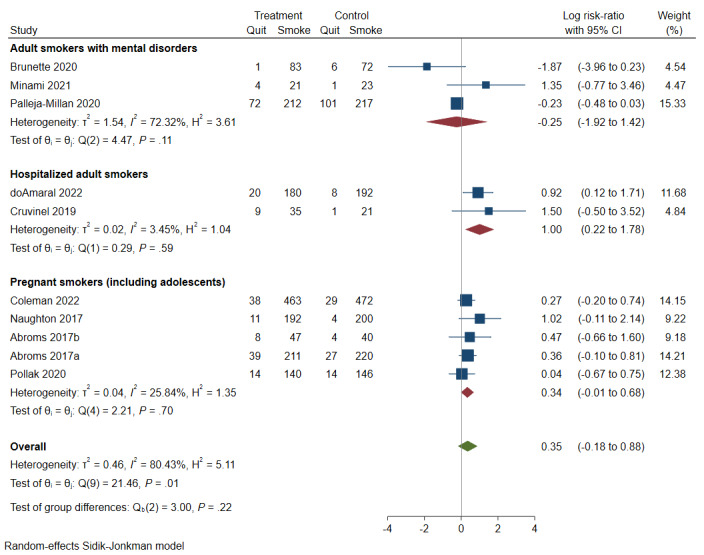
Forest plot of eHealth intervention effects by characteristics of study population (any follow-ups) [40,41,44,47,49,51,57,60-62].

A total of 10 studies reported interventions that targeted comparable populations with special characteristics. Because of the limited number of studies, no further categorization was made based on follow-up, intervention, or control. Hospitalized adult smokers benefited more from eHealth interventions (log RR=1.00, 95% CI 0.22-1.78; *I^2^*=3.45%) than pregnant smokers (log RR=0.34, 95% CI −0.01 to 0.68; *I^2^*=25.84%). Findings on smokers with mental disorders were contradictory and nonsignificant (log RR=−0.25, 95% CI −1.92 to 1.42; *I^2^*=72.32%; Figure 3).

### Publication Bias

**Figure 4:**
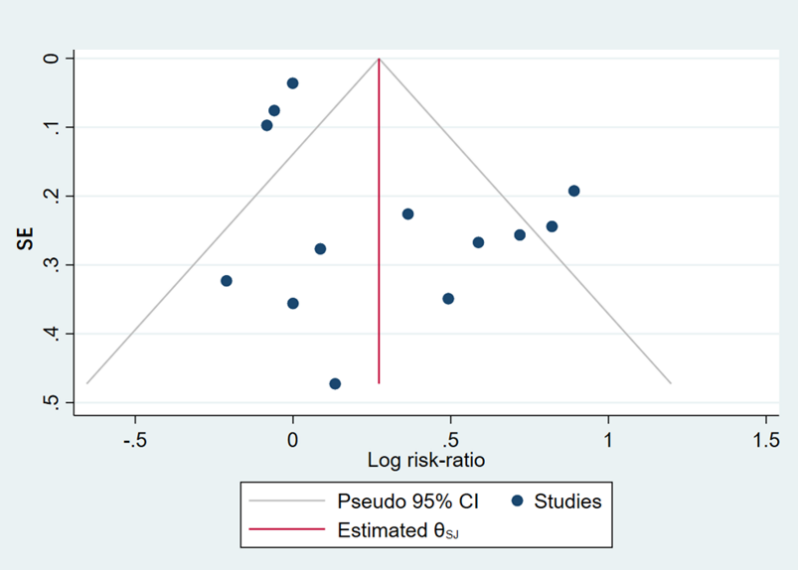
Funnel plot of long-term abstinence results [42,43,46,48,50,53-56,58,59,63,65].

Funnel plots were generated for each pooled result and are presented in Multimedia Appendix 2. Because of the limited number of studies in each group, assessing publication bias is challenging. Therefore, we decided to focus on the subgroup with the largest number of studies (n=13), which included long-term abstinence. Figure 4 displays the funnel plot assessing publication bias among the studies that measured long-term abstinence in adult smokers. Visual inspection of the plot revealed a relatively symmetrical distribution of the included studies, indicating that our study was unlikely to be affected by publication bias.

### Narrative Synthesis

For studies not included in the meta-analysis (n=11), we have summarized the treatment effect on abstinence outcomes in Table 5 because of their distinct features in the study population or interventions. Overall, 2 studies that used multiplatform eHealth intervention or mHealth apps accompanied by SMS text messaging reported a significant increase in abstinence at 3- and 6-month follow-ups [27,28]. A significant increase in abstinence at 6 months was reported in 2 studies using mHealth counseling and SMS messaging accompanied by pharmacotherapy, respectively, as interventions [29,30]. In addition, one study reported a text referral program to refer smokers to cessation services, which may improve cessation outcomes [31]. Finally, acceptance and commitment therapy were suggested to be more effective than US clinical practice guidelines in the context of the mHealth app group [32]. The remaining 5 studies reported no significant differences between intervention and control on cessation outcomes [33-37].

For nonabstinence outcomes, a total of 20 studies reported a reduction in cigarette consumption and user satisfaction (Multimedia Appendix 4 [28,32-34,37-50,52,53,58,60,62,64]). Among the 14 studies that reported cigarette consumption outcomes, only 3 suggested that the intervention can reduce cigarette consumption significantly compared with controls [38-40]. Finally, 19 studies assessed user satisfaction after the intervention, and 18 reported good user satisfaction, whereas the remaining 1 study specifically investigated user adherence to the program [38]. Among the studies that reported high user satisfaction, 8 compared user satisfaction between the intervention and control groups and found significantly higher satisfaction in the intervention arm [28,32,34,41-45].

**Table 5:** Summary of eHealth intervention effects for studies not included in the meta-analysis (n=11).

Study	Intervention vs control	Personalized or interactive	Study population (verification)	Intervention quit, n	Intervention smoking, n	Control quit, n	Control smoking, n	RR^a^ (95% CI)	Summary of outcome
Masaki et al [27]	Integrated eHealth + pharmacotherapy vs control eHealth + pharmacotherapy	Yes	Adult smokers with intention to quit (biochemically validated)	3 months: 215; 6 months: 182; 12 months: 149	3 months: 70; 6 months: 103; 12 months: 136	3 months: 190; 6 months: 145; 12 months: 119	3 months: 97; 6 months: 142; 12 months: 168	3 months: 1.14 (1.03-1.27); 6 months: 1.26 (1.09-1.46); 12 months: 1.26 (1.06-1.50)	Significant increase on cessation outcome at all follow-ups
Fernandes et al [29]	mHealth^b^ counseling vs minimal smoking cessation support	Yes	Adult patients with TB^c^ (self-reported)	6 months: 54	6 months: 26	6 months: 34	6 months: 48	6 months: 1.63 (1.21-2.19)	Significant increase on cessation outcome at 6 months
Weng et al [33]	Tailored SMS text messaging vs nonsmoking or untailored SMS text messaging	Yes	Adult smokers not necessarily have intention to quit (self-reported)	3 months: 50; 6 months: 57; 12 months: 65	3 months: 254; 6 months: 247; 12 months: 239	3 months: 76; 6 months: 81; 12 months: 90	3 months: 299; 6 months: 294; 12 months: 285	3 months: 0.81 (0.59-1.12); 6 months: 0.87 (0.64-1.18); 12 months: 0.89 (0.67-1.18)	No significant increase on cessation outcome at all follow-ups
Graham et al [34]	SMS or app text messaging + web-based vs web-based	Yes	Adult smokers with the intention to quit (self-reported)	3 months: 58; 9 months: 72	3 months: 253; 9 months: 239	3 months: 60; 9 months: 71	3 months: 247; 9 months: 236	3 months: 0.95 (0.69-1.32); 9 months: 1.00 (0.75-1.33)	No significant increase on cessation outcome at all follow-ups
Gram et al [35]	SMS text messaging vs mail	Yes	Adult smokers with the intention to quit (self-reported)	3 months: 319; 6 months: 252	3 months: 1869; 6 months: 1936	3 months: 313; 6 months: 236	3 months: 1834; 6 months: 1911	3 months: 1.00 (0.87-1.16); 6 months: 1.05 (0.89-1.24)	No significant increase on cessation outcome at 3 and 6 months
Danaher et al [28]	mHealth app + SMS text messaging vs computer-assisted intervention	Yes	Adult smokers with the intention to quit (self-reported)	3 months: 131; 6 months: 156	3 months: 502; 6 months: 477	3 months: 73; 6 months: 123	3 months: 565; 6 months: 515	3 months: 1.81 (1.39-2.36); 6 months: 1.28 (1.04-1.58)	Significant increase on cessation outcome at 3 and 6 months
Weng et al [31]	On-site referral vs text-based referral vs minimal smoking cessation support	Yes	Adult smokers with the intention to quit; (biochemically validated)	On-site referral; 3 months: 27; 6 months: 30; text-based referral; 3 months: 23; 6 months: 30	On-site referral; 3 months: 368; 6 months: 365; text-based referral; 3 months: 362; 6 months: 355	3 months: 18; 6 months: 15	3 months: 365; 6 months: 368	On-site referral; 3 months: 1.45 (0.81-2.60); 6 months: 1.9392 (1.06-3.55); text-based referral; 3 months: 1.27 (0.70-2.32); 6 months: 1.99 (1.09-3.64)	No significant increase on cessation outcome at 3 months; significant increase on cessation outcome at 6 months
Kruse et al [36]	SMS or app text messaging + pharmacotherapy vs SMS or app text messaging vs pharmacotherapy vs minimal smoking cessation support	No	Adult smokers with the intention to quit; (biochemically validated)	SMS + NRT^d^; 3 months: 2; SMS; 3 months: 3; NRT; months: 3	SMS + NRT; 3 months: 37; SMS; 3 months: 36; NRT; 3 months: 33	3 months: 1	3 months: 38	SMS + NRT; 3 months: 2.00 (0.19-21.16); SMS; 3 months: 3.00 (0.33-27.6); NRT; 3 months: 3.25 (0.35-29.85)	No significant increase on cessation outcome at 3 months
Vidrine et al [30]	SMS or app text messaging + pharmacotherapy vs phone call + pharmacotherapy vs pharmacotherapy	Yes	Socioeconomically disadvantaged adult smokers with the intention to quit; (biochemically validated)	SMS or app text messaging + pharmacotherapy; 6 months: 28; phone call + pharmacotherapy; 6 months: 19	SMS or app text messaging + pharmacotherapy; 6 months: 160; Phone call + pharmacotherapy; 6 months: 194	6 months: 13	6 months: 210	SMS or app text messaging + pharmacotherapy; 6 months: 2.55 (1.36-4.79); phone call + pharmacotherapy; 6 months: 1.53; (0.78-3.02)	Significant increase on SMS or app text messaging + pharmacotherapy; outcome at 6 months; no significant increase on phone call + pharmacotherapy; outcome at 6 months
White et al [37]	Tailored SMS text messaging vs nonsmoking or untailored SMS text messaging	Yes	Adult smokers with the intention to quit; (self-reported)	3 months: 8	3 months: 93	3 months: 3	3 months: 96	3 months: 2.61 (0.71-9.57)	No significant increase on cessation outcome at 3 months
Bricker et al [32]	mHealth app vs mHealth app based on a different theory	Yes	Adult smokers with the intention to quit; (self-reported)	3 months: 285; 6 months: 359; 12 months: 356	3 months: 929; 6 months: 855; 12 months: 858	3 months: 168; 6 months: 259; 12 months: 302	3 months: 1033; 6 months: 942; 12 months: 899	3 months: 1.68 (1.41-2.00); 6 months: 1.37 (1.19-1.57); 12 months: RR=1.17 (1.02-1.33)	Significant increase in cessation outcome at 3-, 6- and 12-month follow-ups

^a^RR: risk ratio.

^b^mHealth: mobile health.

^c^TB: tuberculosis.

^d^NRT: nicotine replacement therapy.

## Discussion

### Principal Findings

This systematic review included 39 RCTs of eHealth smoking cessation interventions, published between 2017 and 2022 [27-65]. Most of the interventions were classified as mHealth interventions involving mobile SMS or app text messaging and mobile apps. This result suggests that cessation intervention delivery channels have shifted away from internet-based interventions [67] or telephone counseling [15], which were prevalent more than 5 years ago. After pooling the 28 included studies, we found mixed results across studies using different subcategories of eHealth interventions and personalization or interactive status. In addition, the intervention effect on abstinence varied among the study populations. Finally, the meta-analysis indicated that studies using biochemical verification yielded results similar compared with studies only used self-reported abstinence. Among the studies not included in the meta-analysis, approximately half (6/11, 55%) reported a statistically significant positive effect on increasing abstinence. In addition to abstinence, a small number of studies (14/39, 36%) evaluated the effects of eHealth interventions on reduced cigarette consumption. Although almost all studies assessed user satisfaction and revealed a high degree of satisfaction postintervention, less than half of them found significant differences between the intervention and control groups.

This study examined 3 types of mHealth interventions: SMS or app text messaging, stand-alone mHealth apps, and mHealth apps used alongside psycho or pharmacological therapy. These interventions have produced different treatment effects on abstinence. Our findings support a previous review [13] that suggests that SMS or app text messaging is more effective than minimal cessation support in promoting abstinence. However, our study also revealed that increasing the frequency of texting may not have a positive impact on abstinence and may even discourage adherence to the intervention [46]. In addition, a previous review found no evidence that smartphone apps can improve the likelihood of smoking cessation and called for further research in this domain [13]. In contrast, in our study, we found that more recent RCTs testing smartphone apps found an increased chance of abstinence among adult smokers in the short term. Compared with the existing knowledge, we believe this change may be due to the improvements in the overall quality of cessation apps that allow more personalized designs, which subsequently increases acceptability among smokers [68]. Finally, our study found that the use of mHealth apps in conjunction with psycho or pharmacological therapy produces abstinence results similar to those of therapy alone. However, the certainty of the evidence is low, indicating the need for further research in this area.

Our review found a high attrition rate and poor long-term treatment effects among identified mHealth smoking cessation studies, which aligns with previous reviews [12,13]. Previous interventions summarized by Belita and Sidani [69] in their systematic review have also shown high attrition rates ranging from 30% to 50% preinclusion and 10% to 50% postinclusion, with a pooled rate of 10.8% to 77%. Such a high attrition rate can easily render many potential therapies ineffective. Although mHealth technologies can adequately address some logistical factors, such as travel, they are neither important nor significantly associated with attrition rates. Therefore, priority should be given to identifying and comprehending the factors that significantly influence the attrition rate. For example, user satisfaction is an important measure of potential adherence from the perspective of eHealth developers [70]. However, we found that most of the included studies reported high satisfaction rates but still had relatively poor user adherence, as evidenced by the high attrition bias rate (13/39, 33%). This finding suggests that the assessment of user satisfaction alone may not be a reliable factor for predicting adherence, at least in terms of smoking cessation interventions. Researchers and mHealth app developers should consider narrowing the intervention scope based on demographic factors at the design stage and improving personalization based on clinical, behavioral, and health belief factors at the development stage [69]. In fact, the eHealth cessation interventions that managed to achieve the most significant increase in abstinence in our meta-analysis targeted hospitalized smokers (log RR=1.00, 95% CI 0.22-1.78; *I^2^*=3.45%) [40,47]. The success may be due to the good program adherence evidenced by the low attrition rate, thanks to the institutional environment that encourages prohealth behaviors, as well as the intervention material dedicated to this specific population.

To explore the association between personalization status and treatment effect, we pooled studies targeting general smokers according to their personalization or interactive level and found improved abstinence results. Previous research has also suggested that such content can improve medication adherence [71] and eHealth application retention [72], thereby enhancing its effectiveness. It is not surprising that interventions that included some level of personalized or interactive content achieved a significant increase in abstinence rates both in the short and long term. By contrast, studies that lacked any personalized or interactive features showed null effects after pooling. However, caution should be exercised when interpreting these findings because of high within-group heterogeneity.

Finally, we found that studies using either biochemical verification or self-reporting for measuring abstinence showed similar treatment effects. Previous research has produced mixed results on whether self-reported abstinence is a reliable indicator of biochemically verified abstinence. Although some studies have suggested that self-reported quitting is mostly accurate [73], others have found a high proportion of self-reported quitters failing biochemical verification in clinical settings [74]. However, our review brings a new perspective to the debate, suggesting that studies using biochemical verification do not necessarily outperform those using self-reports. We found that the effect sizes of studies using both methods were consistently similar in both the short and long term (Table 4). Although biochemical verification is encouraged by the Society for Research on Nicotine and Tobacco for its scientific rigor, it can be expensive [75]. Our findings support the feasibility of eHealth-based cessation programs, which are scalable to large-scale interventions where biochemical verification is not possible. Nonetheless, given the possibility of false reporting, trials evaluating potential population-level interventions may need to be considered using biochemical verification of smoking populations that are most susceptible to false reports. For instance, a study has recommended using biochemical assessment, preferably with cotinine plasma, in intervention studies and with student populations [76]. Second, there are a variety of biochemical verification methods that target different biomarkers. This diversity has prompted an update of the 2002 Society for Research on Nicotine and Tobacco reports on whether and how to apply biomarker verification to tobacco use and abstinence [75]. Given this complexity, researchers in relevant fields should focus on standardizing currently accepted biochemical verification methods and their cut-off points to improve interstudy compatibility, rather than seeking the most accurate method.

### Strengths and Limitations

This study provides a comprehensive and updated evaluation of the potential role of eHealth interventions in facilitating smoking cessation. Its robustness lies in the inclusion of recent well-funded studies that demonstrate the advancement of digital technology and its accessibility in both high- and lower-income nations (Multimedia Appendix 5 [27-65]). The present review included multiple outcome assessment criteria and treated populations to provide a more holistic evaluation. However, we acknowledge that this study has some limitations. First, our focus was limited to studies published in English in the last 5 years, which means that we may have overlooked relevant research conducted in other languages or low- to middle-income countries. Second, due to the significant heterogeneity of methodological design and outcome verification among studies, not all were suitable for inclusion in the meta-analysis. This limitation led to fewer studies being synthesized, which undermined the certainty of the evidence. In addition, the small sample sizes in some studies resulted in relatively large CIs for effect size estimation, making it difficult to determine a significant effect. Third, despite our attempt to synthesize the intervention effect on special populations and nonabstinence outcomes, such as cigarette consumption, the heterogeneity in outcome measurement among the collected evidence prevented us from drawing any conclusion.

### Future Recommendations

Future studies could standardize the intervention evaluation strategy by following the World Health Organization *Practical Guide to Monitoring and Evaluating Digital Health Interventions* [77] for better comparability between trials. In addition, mobile app development should adopt a human-centered design approach and prioritize improving participant adherence and engagement [78,79] to reduce attrition and achieve better long-term cessation outcomes (≥6 months). Furthermore, research is necessary to understand the effectiveness of eHealth interventions on susceptible populations and intermediate outcomes such as reduction in cigarette consumption.

### Conclusions

The use of eHealth technologies for smoking cessation has gained momentum in recent years. Our review highlighted the timeliness of eHealth interventions, particularly mHealth, in promoting abstinence, although their effectiveness may wane over time. Future studies could benefit from adopting a *learning by doing* approach and incorporating the concept of *human-centered design* to develop personalized intervention designs that address individual smoker needs and reduce attrition, ultimately leading to better long-term abstinence outcomes. In addition, owing to the dynamic nature of eHealth interventions, monitoring and evaluation can be challenging. Standardized evaluation strategies should be implemented to improve interstudy comparability.

## Appendix

Multimedia Appendix 1 Search strategies.

Multimedia Appendix 2 Meta-analysis of forest and funnel plots (random effects).

Multimedia Appendix 3 Summary of eHealth intervention effects (meta studies).

Multimedia Appendix 4 Summary of eHealth intervention effects on nonabstinence outcomes.

Multimedia Appendix 5 Funding information of included randomized controlled trials.

## Abbreviations

GRADE: Grading of Recommendations, Assessment, Development, and Evaluations
mHealth: mobile health
PPA: point prevalence abstinence
PRISMA: Preferred Reporting Items for Systematic Reviews and Meta-Analyses
RCT: randomized controlled trial
RoB 2: version 2 of the Cochrane risk-of-bias tool for randomized trials
RR: risk ratio
