# Table Correction: Effectiveness of eHealth Smoking Cessation Interventions: Systematic Review and Meta-Analysis

**DOI:** 10.2196/56438

**Published:** 2024-02-07

**Authors:** Yichen E Fang, Zhixian Zhang, Ray Wang, Bolu Yang, Chen Chen, Claudia Nisa, Xin Tong, Lijing L Yan

**Affiliations:** 1 Global Health Research Center Duke Kunshan University Kunshan China; 2 Department of Global Health School of Public Health Wuhan University Wuhan China; 3 Division of Social Sciences Duke Kunshan University Kunshan China; 4 Data Science Research Center Duke Kunshan University Kunshan China; 5 Duke Global Health Institute Duke University Durham, NC United States; 6 Institute for Global Health and Development Peking University Beijing China

In “Effectiveness of eHealth Smoking Cessation Interventions: Systematic Review and Meta-Analysis” (J Med Internet Res 2023;25:e45111) the authors noted three errors associated with the cited paper by Bricker et al [1] in Table 5.

In Table 5 row “Bricker et al” of the originally published manuscript, there were several errors.

1) The number of quits and smoking participants at 12-month follow-up in the intervention group was entered incorrectly as "12 months: 256” in “Intervention quit, n” column and “12 months: 856” in “Intervention smoking, n” column. The numbers have been replaced by “12 months: 356” in “Intervention quit, n” column and “12 months: 858” in “Intervention smoking, n”.

This error then led to the following:

2) The incorrect number of “12 months: 0.92 (0.79-1.06)” in the “RR 95% CI” column

3) The incorrect statement of “No significant increase on cessation outcome at 12 months follow-ups; significant increase on cessation outcome at 3- and 6-month follow-ups” in the “Summary of outcome” column

The entry in column “RR 95% CI” has now been corrected to “12 months: RR=1.17 (1.02-1.33)” and the entry in column “Summary of outcome” has now been corrected to “significant increase in cessation outcome at 3-, 6- and 12-month follow-ups”.

The error did not affect any other part of the paper as the error happened during the final formatting stage on the authors’ end and the manuscript content was drafted with the correct numbers.

The correction will appear in the online version of the paper on the JMIR Publications website on February 7, 2024, together with the publication of this correction notice. Because this was made after submission to PubMed, PubMed Central, and other full-text repositories, the corrected article has also been resubmitted to those repositories.
