# Supplementary material for: Effectiveness of eHealth Smoking Cessation Interventions: Systematic Review and Meta-Analysis
Source: J Med Internet Res. 2023 Jul 28;25:e45111. doi: 10.2196/45111 (PMC10422176; doi:10.2196/45111)
Supplement: Multimedia Appendix 1 [file jmir_v25i1e45111_app1.doc]

Search Strategies

1/1/2017~6/30/2022

1. ***Smoking Cessation + Device + Intervention Channel + RCT — PubMed***
2. Smoking Cessation

("Smoking Cessation"[MeSH Terms])

1. Device

("Cell Phone"[MeSH Terms] OR "Smartphone"[MeSH Terms] OR "Computers"[MeSH Terms] OR "Computers, Handheld"[MeSH Terms])

1. Intervention Channel

("Online Systems"[MeSH Terms] OR "Technology"[MeSH Terms] OR "Social Media"[MeSH Terms] OR "Mobile Applications"[MeSH Terms] OR "Text Messaging"[MeSH Terms] OR "Telemedicine"[MeSH Terms] OR "Internet-Based Intervention"[MeSH Terms] OR "Multimedia"[MeSH Terms] OR "Electronic Mail"[MeSH Terms])

1. RCT

((randomized controlled trial[pt]) OR (controlled clinical trial[pt]) OR (randomized[tiab] OR randomised[tiab]) OR (placebo[tiab]) OR (drug therapy[sh]) OR (randomly[tiab]) OR (trial[tiab]) OR (groups[tiab])) NOT (animals[mh] NOT humans[mh])

Reference: <https://libraryguides.mcgill.ca/epib629/rct-filters>

**Combined**

(((("Smoking Cessation"[MeSH Terms])) AND (("Cell Phone"[MeSH Terms] OR "Smartphone"[MeSH Terms] OR "Computers"[MeSH Terms] OR "Computers, Handheld"[MeSH Terms]))) AND (("Online Systems"[MeSH Terms] OR "Technology"[MeSH Terms] OR "Social Media"[MeSH Terms] OR "Mobile Applications"[MeSH Terms] OR "Text Messaging"[MeSH Terms] OR "Telemedicine"[MeSH Terms] OR "Internet-Based Intervention"[MeSH Terms] OR "Multimedia"[MeSH Terms] OR "Electronic Mail"[MeSH Terms]))) AND (((randomized controlled trial[pt]) OR (controlled clinical trial[pt]) OR (randomized[tiab] OR randomised[tiab]) OR (placebo[tiab]) OR (drug therapy[sh]) OR (randomly[tiab]) OR (trial[tiab]) OR (groups[tiab])) NOT (animals[mh] NOT humans[mh]))

1. ***Smoking Cessation + Device + Intervention Channel + RCT — Psycinfo***
2. Smoking Cessation

(DE "Smoking Cessation")

1. Device

(DE "Mobile Phones") OR (DE "Mobile Devices" OR DE "Telephone Systems" OR DE "Smartphones" OR DE "Mobile Applications" OR DE "Mobile Assessment" OR DE "Smartphone Use" OR DE "Text Messaging" OR DE "Wireless Technologies") OR (DE "Smartphones" OR DE "Mobile Phones" OR DE "Smartphone Use") OR (DE "Computers") OR (DE "Tablet Computers" OR DE "Mobile Devices" OR DE "Digital Gaming" OR DE "Mobile Applications")

1. Intervention Channel

(DE "Online Therapy" OR DE "Telemedicine" OR DE "Computer Mediated Communication" OR DE "Internet" OR DE "Telecommunications Media") OR (DE "Mobile Applications" OR DE "Computer Applications" OR DE "Mobile Technology" OR DE "Digital Gaming" OR DE "Electronic Communication" OR DE "Mobile Health" OR DE "Mobile Learning") OR (DE "Social Media") OR (DE "Text Messaging") OR (DE "Digital Interventions") OR (DE "Multimedia")

1. RCT

("double-blind" OR "random* assigned" OR control)

Reference: <https://work.cochrane.org/psycinfo>

**Combined**

(DE "Smoking Cessation") AND ( (DE "Mobile Phones") OR (DE "Mobile Devices" OR DE "Telephone Systems" OR DE "Smartphones" OR DE "Mobile Applications" OR DE "Mobile Assessment" OR DE "Smartphone Use" OR DE "Text Messaging" OR DE "Wireless Technologies") OR (DE "Smartphones" OR DE "Mobile Phones" OR DE "Smartphone Use") OR (DE "Computers") OR (DE "Tablet Computers" OR DE "Mobile Devices" OR DE "Digital Gaming" OR DE "Mobile Applications") ) AND ( (DE "Online Therapy" OR DE "Telemedicine" OR DE "Computer Mediated Communication" OR DE "Internet" OR DE "Telecommunications Media") OR (DE "Mobile Applications" OR DE "Computer Applications" OR DE "Mobile Technology" OR DE "Digital Gaming" OR DE "Electronic Communication" OR DE "Mobile Health" OR DE "Mobile Learning") OR (DE "Social Media") OR (DE "Text Messaging") OR (DE "Digital Interventions") OR (DE "Multimedia") ) AND ( ("double-blind" OR "random* assigned" OR control) )

1. ***Smoking Cessation + Device + Intervention Channel + RCT — Embase***
2. Smoking Cessation

('smoking cessation'/exp OR 'smoking cessation program'/exp)

1. Device

('mobile phone'/exp OR 'computer'/exp)

1. Intervention Channel

('mobile phone'/exp OR 'computer'/exp OR 'online system'/exp OR 'technology'/exp OR 'social media'/exp OR 'mobile application'/exp OR 'text messaging'/exp OR 'telemedicine'/exp OR 'web-based intervention'/exp OR 'multimedia'/exp OR 'e-mail'/exp)

1. RCT

('crossover procedure':de OR 'double-blind procedure':de OR 'randomized controlled trial':de OR 'single-blind procedure':de OR (random* OR factorial* OR crossover* OR cross NEXT/1 over* OR placebo* OR doubl* NEAR/1 blind* OR singl* NEAR/1 blind* OR assign* OR allocat* OR volunteer*):de,ab,ti)

Reference: <https://aub.edu.lb.libguides.com/c.php?g=329862&p=3023731>

**Combined**

#1 AND #2 AND #3 AND #4 AND [2017-2022]/py

1. ***Smoking Cessation + Device + Intervention Channel + RCT — Cochrane Library***
2. Smoking Cessation

#1 MeSH descriptor: [Smoking Cessation] explode all trees 4435

1. Device

#2 MeSH descriptor: [Cell Phone] explode all trees 2283

#3 MeSH descriptor: [Smartphone] explode all trees 600

#4 MeSH descriptor: [Computers] explode all trees 2083

#5 MeSH descriptor: [Computers, Handheld] explode all trees 966

1. Intervention Channel

#6 MeSH descriptor: [Online Systems] explode all trees 209

#7 MeSH descriptor: [Technology] explode all trees 6403

#8 MeSH descriptor: [Social Media] explode all trees 271

#9 MeSH descriptor: [Mobile Applications] explode all trees 1087

#10 MeSH descriptor: [Text Messaging] explode all trees 1140

#11 MeSH descriptor: [Telemedicine] explode all trees 3253

#12 MeSH descriptor: [Internet-Based Intervention] explode all trees 357

#13 MeSH descriptor: [Multimedia] explode all trees 255

#14 MeSH descriptor: [Electronic Mail] explode all trees 363

1. RCT

Results in the Cochrane Central Register of Controlled Trials (CENTRAL)

**Combined**

(#1) AND (#2 OR #3 OR #4 OR #5) AND (#6 OR #7 OR #8 OR #9 OR #10 OR #11 OR #12 OR #13 OR #14)
