# Supplementary material for: Effectiveness of eHealth Smoking Cessation Interventions: Systematic Review and Meta-Analysis
Source: J Med Internet Res. 2023 Jul 28;25:e45111. doi: 10.2196/45111 (PMC10422176; doi:10.2196/45111)
Supplement: Multimedia Appendix 2 [file jmir_v25i1e45111_app2.doc]

**Random-Effects (Hartung-Knapp-Sidik-Jonkman)**

**Short-term Abstinence (3-months)**

1. High-Frequency SMS/App text messaging vs Low-Frequency SMS/App text messaging:
2. SMS/App text messaging vs minimal smoking cessation support
3. mHealth app vs less intensive smoking cessation support
4. Biochemically verified outcome vs Self-reported outcome
5. Personalized vs Not Personalized/Interactive


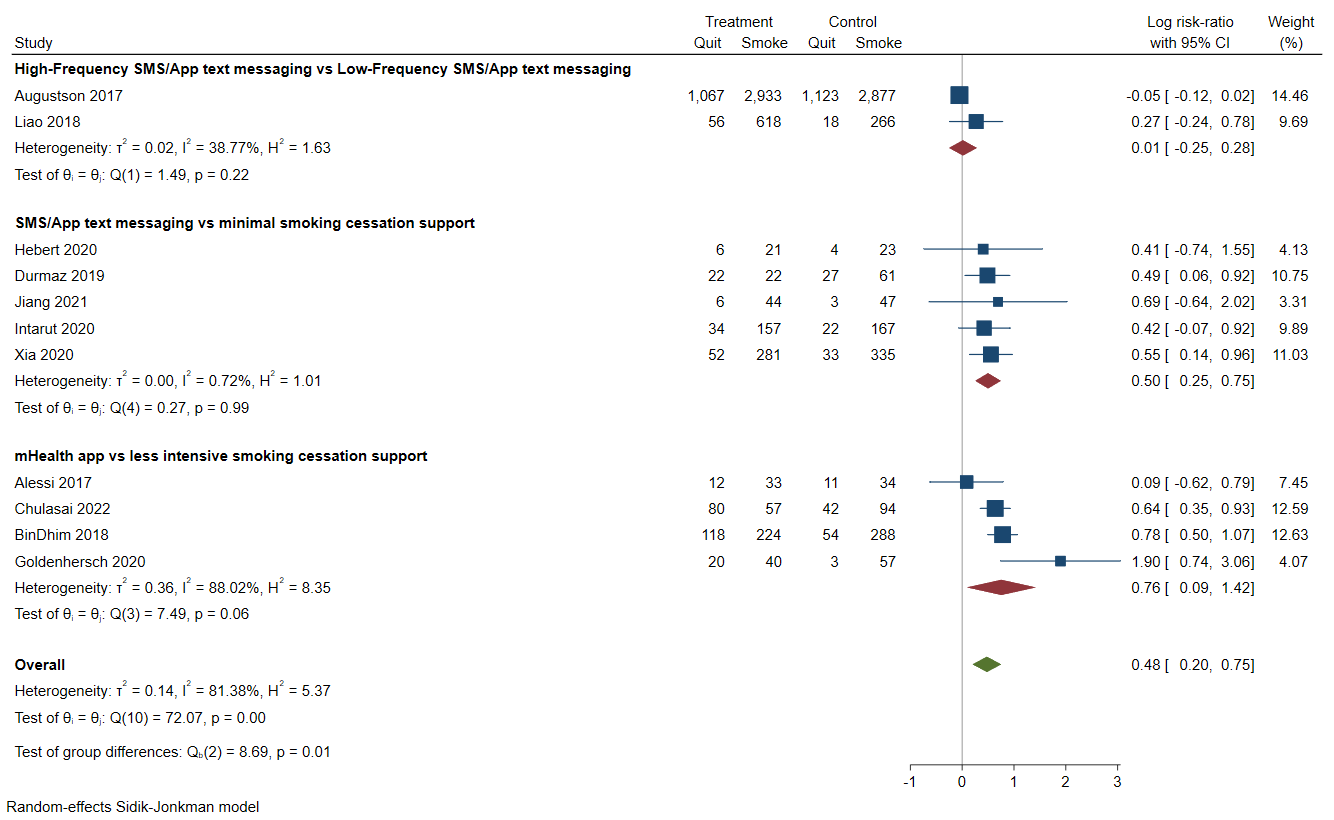


**Figure 1.** Forest Pot of Short-term eHealth Intervention Effects by Characteristics of Intervention and Control Group


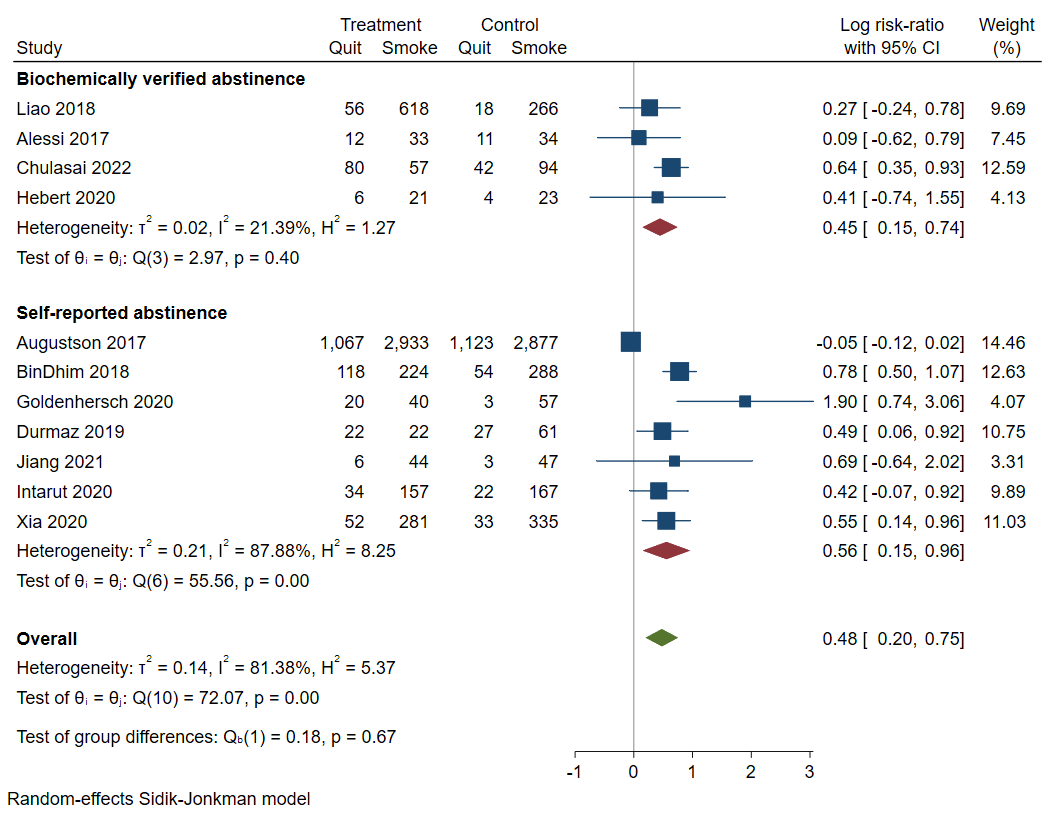


**Figure 2.** Forest Pot of Short-term eHealth Intervention Effects by Outcome Verification


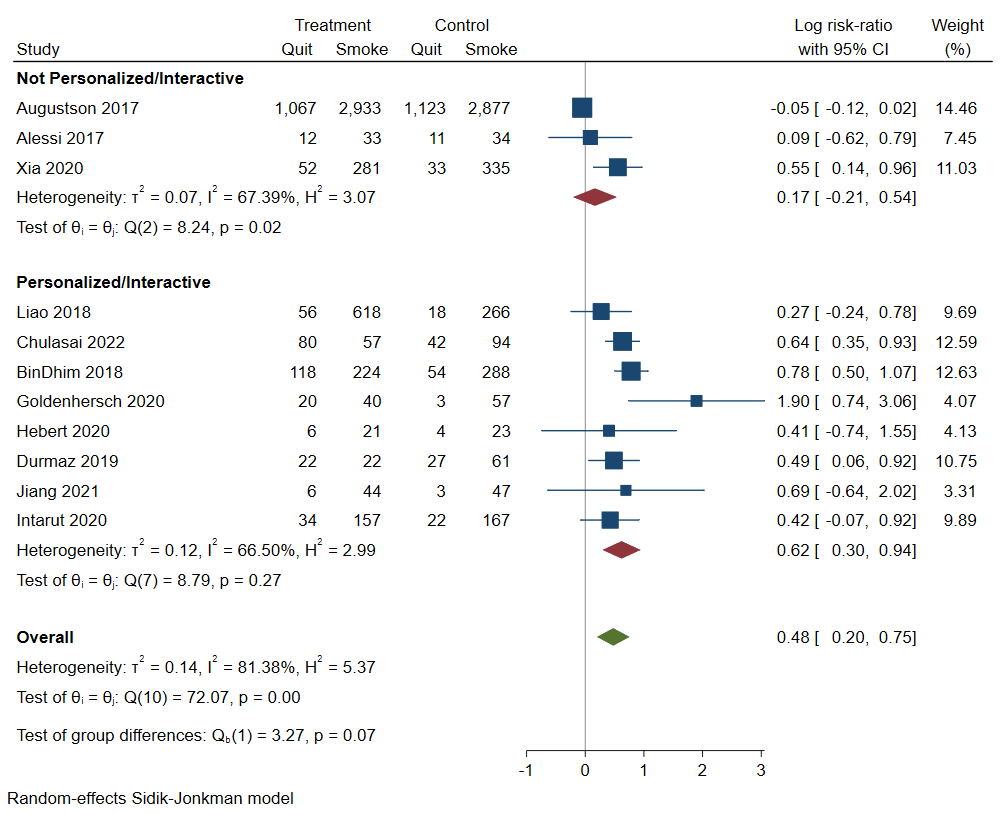


**Figure 3.** Forest Pot of Short-term eHealth Intervention Effects by Personalization


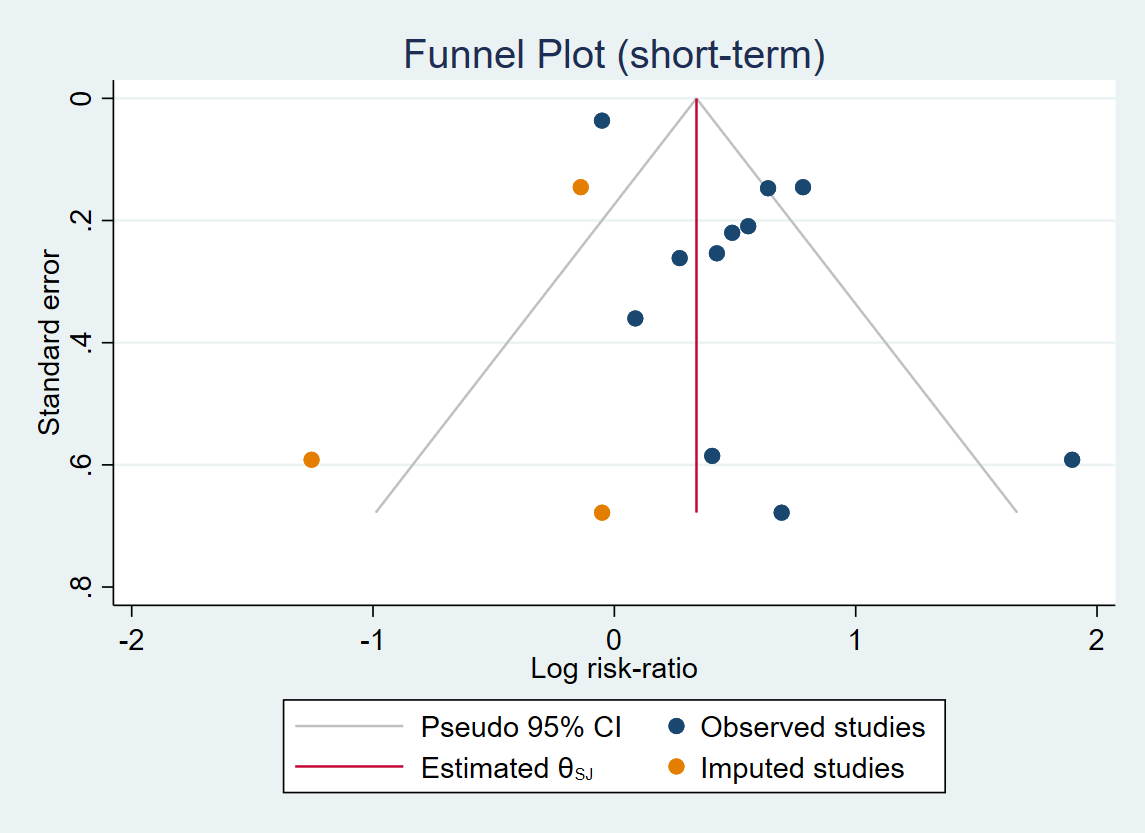


**Figure 4.** Funnel Plot of Short-term Abstinence Results after a Nonparametric Trim and Fill Analysis of Publication Bias

**Long-term Abstinence (6-months)**

1. High-Frequency SMS/App text messaging vs Low-Frequency SMS/App text messaging:
2. SMS/App text messaging vs minimal smoking cessation support
3. mHealth app + psycho/pharmacological therapy vs psycho/pharmacological therapy
4. mHealth app vs less intensive smoking cessation support
5. Biochemically verified outcome
6. Self-reported outcome


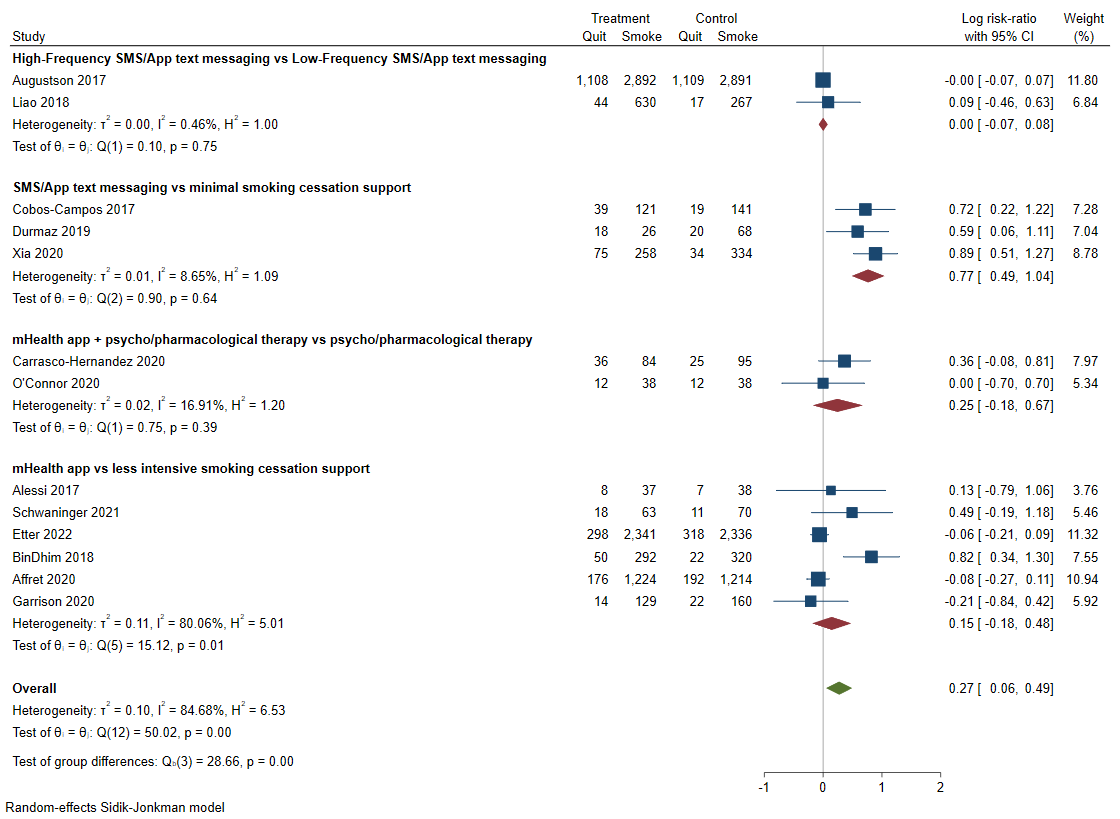


**Figure 5.** Forest Plot of Long-term eHealth Intervention Effects by Characteristics of Intervention and Control Group


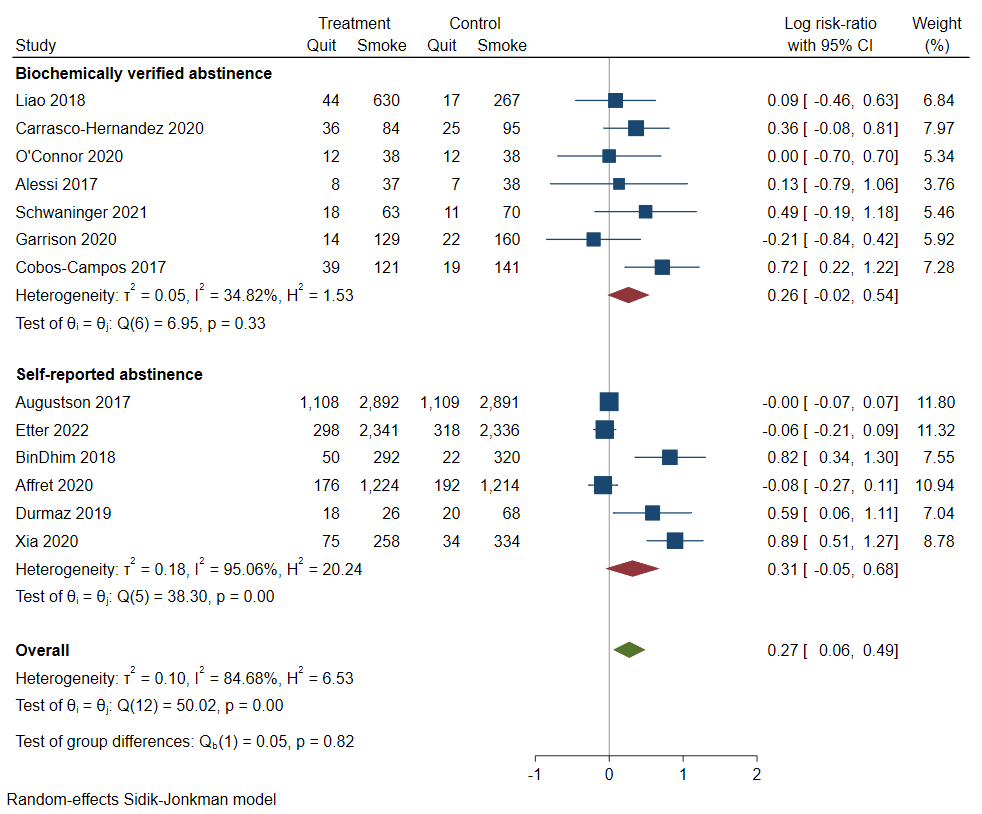


**Figure 6.** Forest Plot of Long-term eHealth Intervention Effects by Outcome Verification


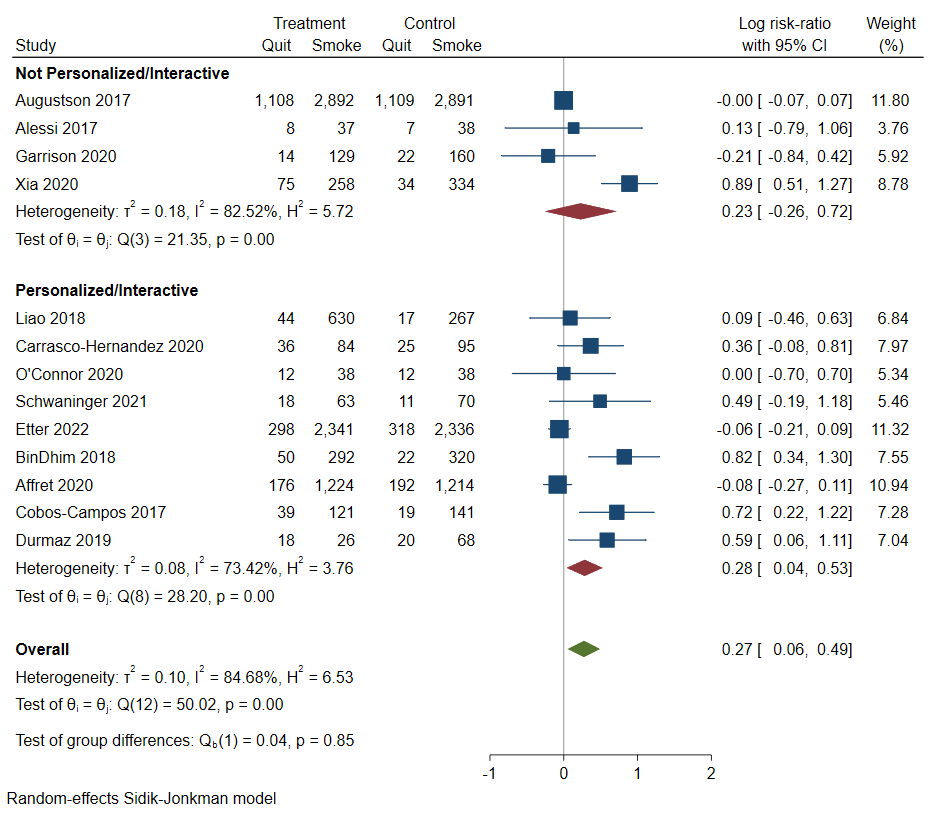


**Figure 7.** Forest Plot of Long-term eHealth Intervention Effects by Personalization


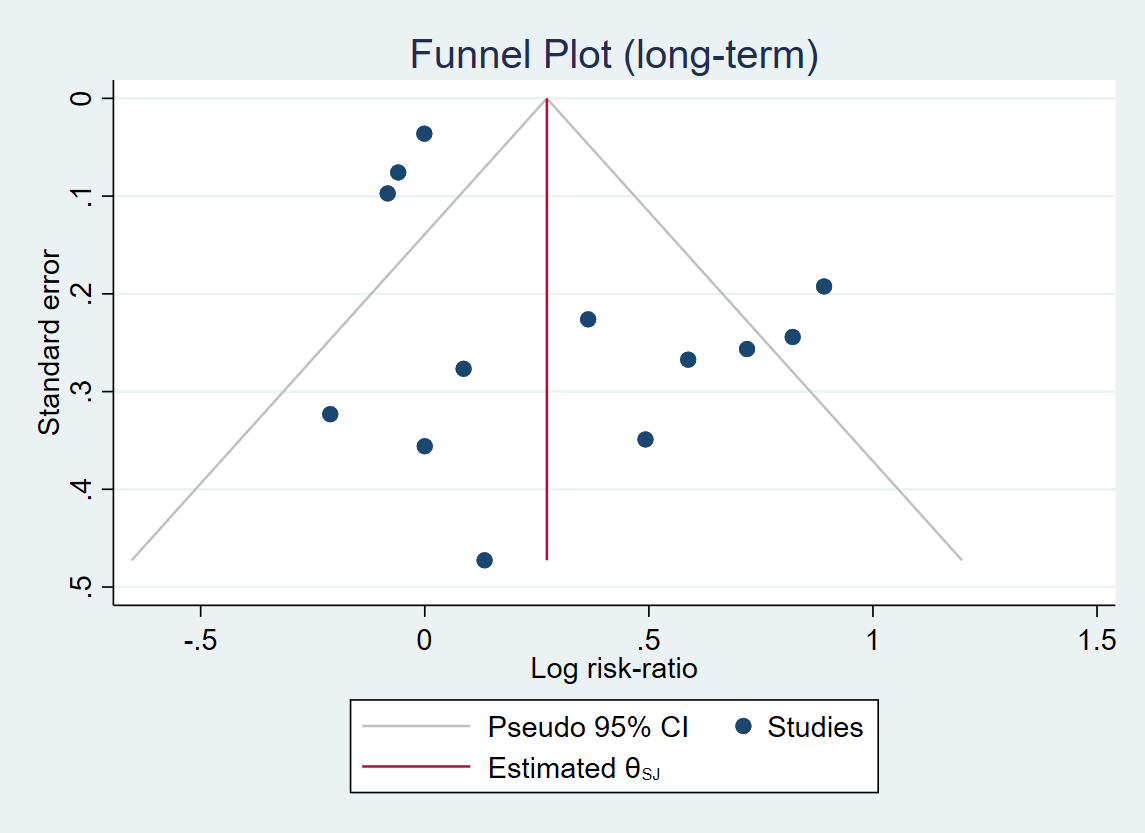


**Figure 8.** Funnel Plot of Short-term Abstinence Results

**eHealth interventions Abstinence Results of Special Population (Any Follow-up)**

1. Adult smokers with mental disorders
2. Hospitalized adult smokers
3. Pregnant smokers (including adolescents)


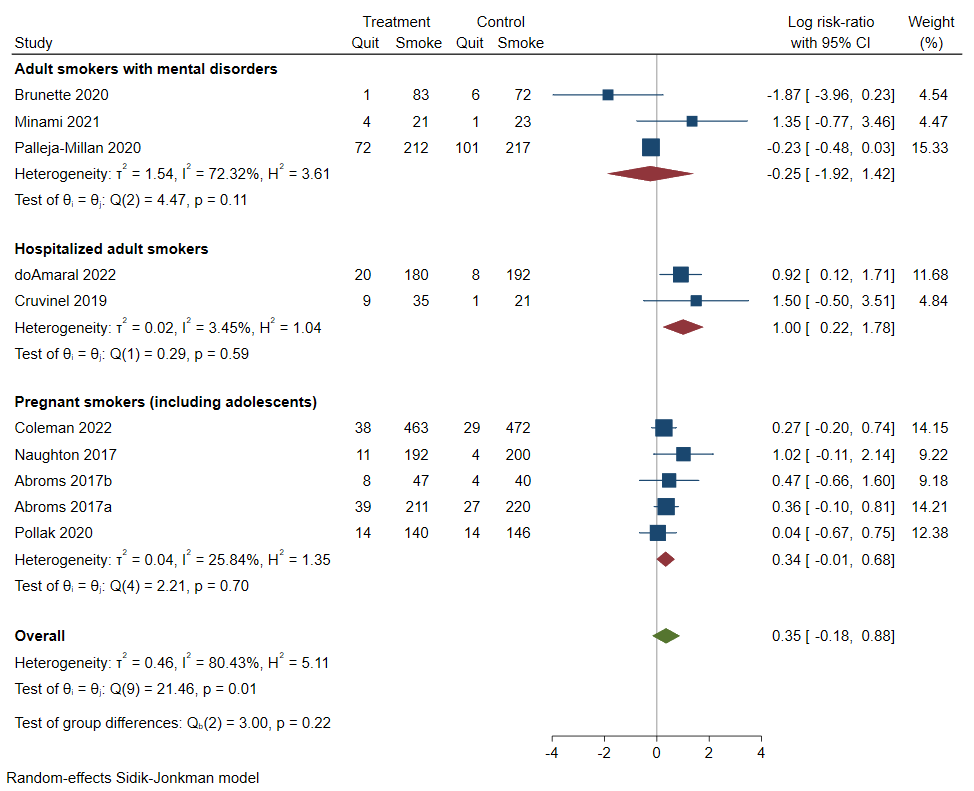


**Figure 9.** Forest Plot of eHealth Intervention Effects by Characteristics of Study Population (Any Follow-up)


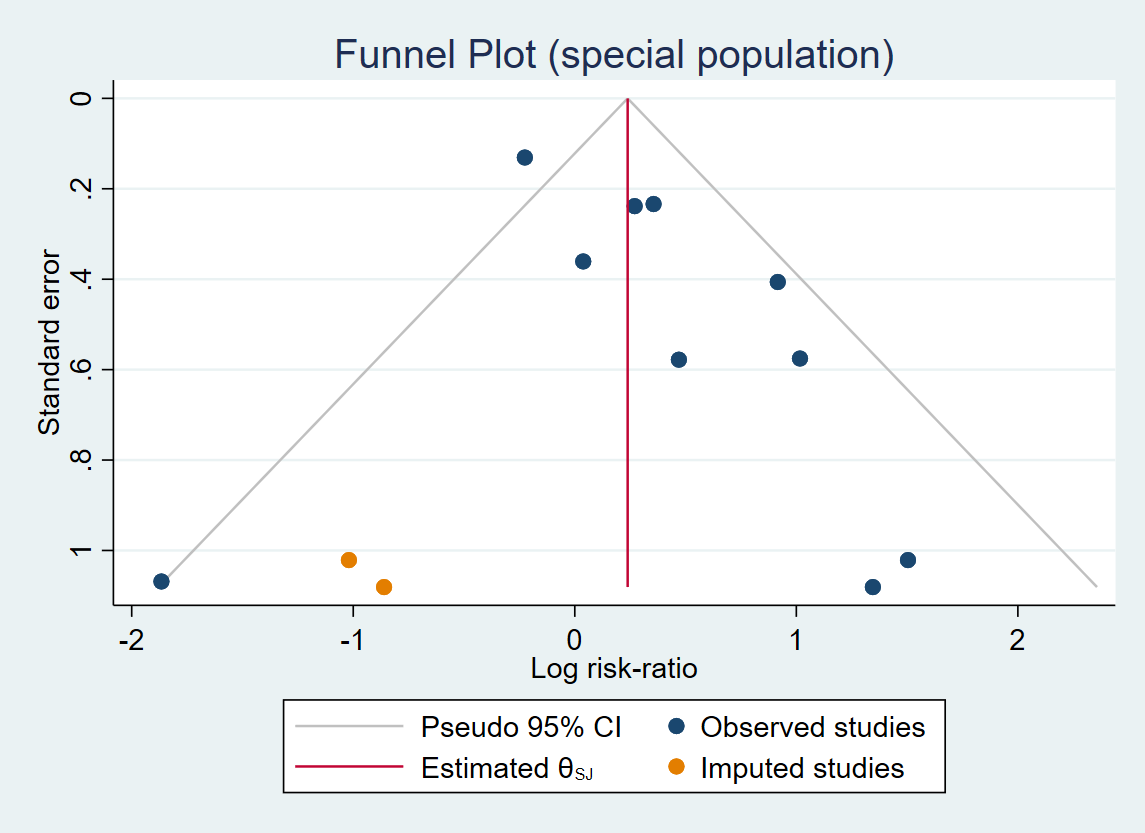


**Figure 4.** Funnel plot of Special Population Abstinence Results after a Nonparametric Trim and Fill Analysis of Publication Bias
