# Supplementary material for: Effectiveness of eHealth Smoking Cessation Interventions: Systematic Review and Meta-Analysis
Source: J Med Internet Res. 2023 Jul 28;25:e45111. doi: 10.2196/45111 (PMC10422176; doi:10.2196/45111)
Supplement: Multimedia Appendix 3 [file jmir_v25i1e45111_app3.docx]

**Table 1.** Summary of eHealth intervention effects for studies included in the meta-analysis (n=28)

| **Study** | **Intervention vs. Control** | **Personalized/Interactive** | **Study Population (Verification)** | **Intervention Quit, n** | **Intervention Smoking, n** | **Control Quit, n** | **Control Smoking, n** | **Risk ratio (95% CI)** | **Summary of Outcome** |
| --- | --- | --- | --- | --- | --- | --- | --- | --- | --- |
| Xia 2020 | SMS/App text messaging vs Video vs minimal smoking cessation support | No | Adult smokers - expectant fathers voluntary recruited | SMS/App text messaging:  3-months: 52  6-months: 75  Video: 3-months: 36  6-months: 48 | SMS/App text messaging:  3-months: 281  6-months: 258  Video:  3-months: 286  6-months: 274 | 3-months: 33  6-months: 34 | 3-months: 335  6-months: 334 | 3-months（SMS）: 1.7414 (1.16-2.62)  6-months（SMS）: 2.4377 (1.67-3.55)  3-months (video): 1.25 (0.80-1.95)  6-months (video): 1.61 (1.07-2.44) | Significant increase on cessation outcome at all follow-ups, except for 2-months (video) |
| Alessi 2017 | mHealth app vs less intensive smoking cessation support | No | Adult smokers with the intention to quit | 3-months: 12  6-months: 8 | 3-months: 33  6-months: 37 | 3-months: 11  6-months: 7 | 3-months: 34  6-months: 38 | 3-months: 1.09 (0.54 to 2.21)  6-months: 1.14 (0.45 to 2.89) | No significant increase on cessation outcome at follow-ups |
| Brunette 2020 | Web-based vs nonactive control | Yes | Adult smokers with schizophrenia | 6-months: 1 | 6-months: 83 | 6-months: 6 | 6-months: 72 | 6-months: 0.15 (0.02 to 1.26) | No significant increase on cessation outcome at all follow-ups |
| Hebert 2020 | SMS/App text messaging vs mHealth app vs minimal smoking cessation support | Yes | Adult smokers with the intention to quit | SMS/App text messaging  3-months: 6  mHealth app  3-months: 4 | SMS/App text messaging  3-months: 21  mHealth app  3-months: 23 | 3-months: 4 | 3-months:23 | SMS: 3-months: 1.50 (0.48 to 4.72)  mHealth app  3-months: 1.00 (0.28 to 3.59) | No significant increase on cessation outcome at all follow-ups |
| Carrasco-Hernandez 2020 | mHealth app + Psychopharmacological therapy vs Psychopharmacological therapy | Yes | Adult smokers with the intention to quit | 3-months: 58  6-months: 36  12-months: 34 | 3-months: 62  6-months: 84  12-months: 86 | 3-months: 50  6-months: 25  12-months: 19 | 3-months: 70  6-months: 95  12-months: 101 | 3-months: 1.16 (0.88 to 1.54)  6-months: 1.44 (0.92 to 2.24)  12-months: 1.79 (1.08 to 2.95) | No significant increase on cessation outcome at 3-months and 6-months, significant increase at 12-months |
| Goldenhersch 2020 | mHealth app vs less intensive smoking cessation support | Yes | Adult smokers with the intention to quit | 3-months: 20 | 3-months: 40 | 3-months: 3 | 3-months: 57 | 3-months: 6.67（2.09 to 21.26） | Significant increase on cessation outcome at 3-months and 6-months |
| Minami 2021 | mHealth app vs less intensive smoking cessation support | No | Adults with mood disorder and intention to quit | 3-months: 1 | 3-months: 23 | 3-months: 4 | 3-months: 21 | 3-months: 0.26 (0.03 to 2.17) | No significant increase on cessation outcome at follow-ups |
| Chulasai 2022 | mHealth app vs less intensive smoking cessation support | Yes | Adult smokers with the intention to quit | 3-months: 80 | 3-months: 57 | 3-months: 42 | 3-months: 94 | 1.64 (0.83 to 3.24) | No significant increase on cessation outcome at follow-ups |
| Schwaninger 2021 | mHealth app vs less intensive smoking cessation support | Yes | Adult smokers with the intention to quit | 6-months: 18 | 6-months: 63 | 6-months: 11 | 6-months: 70 | 1.64 (0.83 to 3.24) | No significant increase on cessation outcome at follow-ups |
| Coleman 2022 | SMS/App text messaging vs minimal smoking cessation support | Yes | Pregnant smokers (including adolescents) | 6-months: 57 | 6-months: 247 | 6-months: 81 | 6-months: 294 | 0.87 (0.64 to 1.18) | No significant increase on cessation outcome at follow-ups |
| doAmaral 2022 | SMS/App text messaging vs minimal smoking cessation support | Yes | Hospitalized adult smokers | 3-months: 20 | 3-months: 180 | 3-months: 8 | 3-months: 192 | 2.5(1.13 to 5.54) | Significant increase on cessation outcome at 3-months follow-ups |
| Etter 2022 | mHealth app vs less intensive smoking cessation support | Yes | Adult smokers with the intention to quit | 6-months: 298 | 6-months: 2341 | 6-months: 318 | 6-months: 2336 | 0.94(0.81 to 1.09) | No significant increase on cessation outcome at follow-ups |
| Naughton 2017 | SMS/App text messaging vs minimal smoking cessation support | Yes | Pregnant smokers (including adolescents) | 3-months: 11  6-months: 15  12-months: 8 | 3-months: 192  6-months: 188  12-months: 195 | 3-months: 4  6-months: 9  12-months: 2 | 3-months:200  6-months: 195  12-months: 202 | 3-months: 2.76 (0.89 to 8.54)  6-months: 1.67 (0.75 to 3.74)  12-months: 4.02 (0.86 to 18.70) | No significant increase on cessation outcome at follow-ups |
| Cobos-Campos 2017 | SMS/App text messaging vs minimal smoking cessation support | Yes | Adult smokers with the intention to quit | 6-months: 39  12-months: 26 | 6-months: 121  12-months: 134 | 6-months: 19  12-months: 9 | 6-months: 141  12-months: 151 | 6-months: 2.05 (1.24 to 3.40)  12-months: 2.89 (1.40 to 5.97) | Significant increase on cessation outcome at follow-ups |
| Cruvinel 2019 | SMS/App text messaging vs minimal smoking cessation support | Yes | Hospitalized adult smokers | 6-months:9 | 6-months:25 | 6-months:1 | 6-months:21 | 5.82 (0.79 to 42.82) | No significant increase on cessation outcome at follow-ups |
| BinDhim 2018 | mHealth app vs less intensive smoking cessation support | Yes | Adult smokers with the intention to quit | 3-months: 118  6-months: 50 | 3-months: 224  6-months: 292 | 3-months: 54  6-months: 22 | 3-months: 288  6-months: 320 | 3-months: 2.19 (1.64 to 2.91)  6-months: 2.27 (1.41 to 3.67) | Significant increase on cessation outcome at follow-ups |
| Durmaz 2019 | SMS/App text messaging vs minimal smoking cessation support | Yes | Adult smokers with the intention to quit | 3-months: 22  6-months: 18 | 3-months: 22  6-months: 26 | 3-months: 27  6-months: 20 | 3-months: 61  6-months: 68 | 3-months: 1.63 (1.06 to 2.50）  6-months: 1.80 (1.07 to 3.04) | Significant increase on cessation outcome at follow-ups |
| Abroms 2017 | SMS/App text messaging vs minimal smoking cessation support | No | Pregnant smokers (including adolescents) | 3-months: 8 | 3-months: 47 | 3-months: 4 | 3-months: 40 | 3-months: 1.60 (0.52 to 4.97) | No significant increase on cessation outcome at follow-ups |
| Jiang 2021 | SMS/App text messaging vs minimal smoking cessation support | Yes | Adult smokers with the intention to quit | 3-months: 6 | 3-months: 44 | 3-months: 3 | 3-months: 47 | 2.00(0.53 to 7.56) | No significant increase on cessation outcome at follow-ups |
| Intarut 2020 | SMS/App text messaging vs minimal smoking cessation support | Yes | Adult smokers with the intention to quit | 3-months: 34 | 3-months: 157 | 3-months: 22 | 3-months: 167 | 1.53 (0.93 to 2.51) | No significant increase on cessation outcome at follow-ups |
| Affret 2020 | mHealth app vs less intensive smoking cessation support | Yes | Adult smokers with the intention to quit | 6-months: 176 | 6-months: 1224 | 6-months: 192 | 6-months: 1214 | 0.92 (0.76 to 1.11) | No significant increase on cessation outcome at follow-ups |
| Garrison 2020 | mHealth app vs less intensive smoking cessation support | No | Adult smokers with the intention to quit | 6-months: 14 | 6-months:129 | 6-months:22 | 6-months: 160 | 0.81 (0.43 to 1.53) | No significant increase on cessation outcome at follow-ups |
| O'Connor 2020 | mHealth app + psychotherapy vs psychotherapy vs less intensive smoking cessation support | Yes | Adult smokers with the intention to quit | 6-months:  mHealth app + psychotherapy: 12  psychotherapy: 12 | 6-months:  mHealth app + psychotherapy: 38  psychotherapy：38 | 6-months:10 | 6-months: 40 | mHealth app + psychotherapy: 1.20 (0.57 to 2.52)  psychotherapy: 1.20 (0.57 to 2.52) | No significant increase on cessation outcome at follow-ups |
| Palleja-Millan 2020 | mHealth app vs less intensive smoking cessation support | Yes | Adult smokers with mental disorders | 3-months: 72  12-months: 39 | 3-months: 212  12-months: 245 | 3-months: 101  12-months: 60 | 3-months:217  12-months: 258 | 3-months: 0.80 (0.62 to 1.03)  12-months: 0.73 (0.50 to 1.05) | No significant increase on cessation outcome at follow-ups |
| Abroms 2019 | Tailored SMS text messaging vs nonsmoking/untailored SMS text messaging | Yes | Pregnant smokers (including adolescents) | 3-months: 39 | 3-months: 211 | 3-months: 27 | 3-months: 220 | 1.43 (0.90 to 2.26) | No significant increase on cessation outcome at follow-ups |
| Pollak 2020 | SMS/App text messaging + alet texts vs SMS/App text messaging | No | Pregnant smokers (including adolescents) | 3-months: 0 | 3-months: 157 | 3-months: 15 | 3-months: 148 | 0.03 (0.00 to 0.55) | No significant increase on cessation outcome at follow-ups |
| Augustson 2017 | High-Frequency SMS/App text messaging vs Low-Frequency SMS/App text messaging | No | Adult smokers with the intention to quit | 3-months:1067  6-months:1108 | 3-months:2933  6-months:2892 | 3-months:1123  6-months:1109 | 3-months:2877  6-months:2891 | 3-months: 0.95 (0.88 to 1.02)  6-months: 1.00 (0.93 to 1.07) | No significant increase on cessation outcome at follow-ups |
| Liao 2018 | High-Frequency SMS/App text messaging vs Low-Frequency SMS/App text messaging vs nonsmoking/untailored SMS text messaging | Yes | Adult smokers with the intention to quit | High-Frequency SMS/App text messaging  3-months:  56  6-months:  44  Low-Frequency SMS/App text messaging  3-months:  18  6-months:  17 | High-Frequency SMS/App text messaging  3-months:  618  6-months:  630  Low-Frequency SMS/App text messaging  3-months:  266  6-months:  267 | 3-months: 9  6-months:8 | 3-months: 402  6-months: 403 | High-Frequency SMS/App text messaging  3-months:  3.79 (1.90 to 7.59)  6 months: 3.35 (1.59 to 7.05)  Low-Frequency SMS/App text messaging  3-months:  2.89 (1.32 to 6.35)  6-months: 3.08 (1.35 to 7.03) | Significant increase on cessation outcome at follow-ups |
