# Supplementary material for: Effectiveness of eHealth Smoking Cessation Interventions: Systematic Review and Meta-Analysis
Source: J Med Internet Res. 2023 Jul 28;25:e45111. doi: 10.2196/45111 (PMC10422176; doi:10.2196/45111)
Supplement: Multimedia Appendix 4 [file jmir_v25i1e45111_app4.docx]

*Table 1.* Summary of eHealth intervention effects on non-abstinence outcomes

| **Study or subgroup** | **Intervention vs. Control** | **Study Population** | **Cigarette consumption (Intervention vs control)** | **Satisfaction**  **(Intervention vs control)** |
| --- | --- | --- | --- | --- |
| **Studies reported 2 non-abstinence outcomes** | | | | |
| Abroms 2017b | SMS/App text messaging vs minimal smoking cessation support | Pregnant smokers (including adolescents) | No significant difference | Significant higher satisfaction |
| Danaher 2019 | mHealth app vs computer assisted intervention | Adult smokers with the intention to quit | No significant difference | Significant higher satisfaction |
| Etter 2022 | mHealth app vs less intensive smoking cessation support | Adult smokers with the intention to quit | No significant difference | Significant higher satisfaction |
| White 2020 | Tailored SMS text messaging vs nonsmoking/untailored SMS text messaging | Adult smokers with the intention to quit | No significant difference | High satisfaction in both groups |
| O'Connor 2020 | mHealth app + psychotherapy vs psychotherapy vs less intensive smoking cessation support | Adult smokers with the intention to quit | No significant difference | Significant higher satisfaction |
| Brunette 2020 | Brief, web-based interventions to motivate smokers with schizophrenia: Randomized controlled trial | Adult smokers with schizophrenia | No significant difference | Significant higher satisfaction |
| Cruvinel 2019 | SMS/App text messaging vs minimal smoking cessation support | Hospitalized adult smokers | No significant difference | High satisfaction (no comparison) |
| Minami 2021 | mHealth app vs less intensive smoking cessation support | Adults with mood disorder and intention to quit | No significant difference | High satisfaction (no comparison) |
| Goldenhersch 2020 | mHealth app vs less intensive smoking cessation support | Adult smokers with the intention to quit | Significant decrease compared to control | Moderate adherent (no comparison) |
| Chulasai 2022 | mHealth app vs less intensive smoking cessation support | Adult smokers with the intention to quit | Significant decrease compared to control | High satisfaction (no comparison) |
| **Studies reported 1 non-abstinence outcome** | | | | |
| Alessi 2017 | mHealth app vs less intensive smoking cessation support | Adult smokers with the intention to quit | No significant difference | NA |
| doAmaral 2022 | SMS/App text messaging vs minimal smoking cessation support | Hospitalized adult smokers | Significant decrease compared to control | NA |
| Intarut 2020 | SMS/App text messaging vs minimal smoking cessation support | Adult smokers with the intention to quit | No significant difference | NA |
| Naughton 2017 | SMS/App text messaging vs minimal smoking cessation support | Pregnant smokers (including adolescents) | NA | Moderate satisfaction (no comparison) |
| Graham 2022 | SMS/App text messaging + web-based vs web-based | Adult smokers with the intention to quit | NA | Significant higher satisfaction |
| Carrasco-Hernandez 2020 | mHealth app + Psychopharmacological therapy vs Psychopharmacological therapy | Adult smokers with the intention to quit | NA | High satisfaction (no comparison) |
| Bricker 2020 | mHealth app vs mHealth app based on a different theory | Adult smokers with the intention to quit | NA | Significant higher satisfaction |
| Cobos-Campos 2017 | SMS/App text messaging vs minimal smoking cessation support | Adult smokers with the intention to quit | NA | High satisfaction (no comparison) |
| Hebert 2020 | SMS/App text messaging vs mHealth app vs minimal smoking cessation support | Adult smokers with the intention to quit | NA | Significant higher satisfaction |
| Augustson 2017 | High-Frequency SMS/App text messaging vs Low-Frequency SMS/App text messaging | Adult smokers with the intention to quit | NA | High satisfaction (no comparison) |
| Pollak 2020 | SMS/App text messaging + alet texts vs SMS/App text messaging | Pregnant smokers (including adolescents) | NA | High satisfaction (no significant difference) |
| Garrison 2020 | mHealth app vs less intensive smoking cessation support | Adult smokers with the intention to quit | No significant difference | NA |
| Jiang 2021 | SMS/App text messaging vs minimal smoking cessation support | Adult smokers with the intention to quit | NA | Moderate satisfaction (no comparison) |
| Weng 2022 | Tailored SMS text messaging vs nonsmoking/untailored SMS text messaging | Adult smokers (not necessarily have intention to quit) | No significant difference | NA |
